# Supplementary material for: Activity and retinoic acid drive hair cell spatial patterning in the zebrafish utricle
Source: Development. 2026 Mar 23;153(6):dev205363. doi: 10.1242/dev.205363 (PMC13196668; doi:10.1242/dev.205363)
Supplement: Supplementary file 1 [file develop-153-205363-s1.pdf]

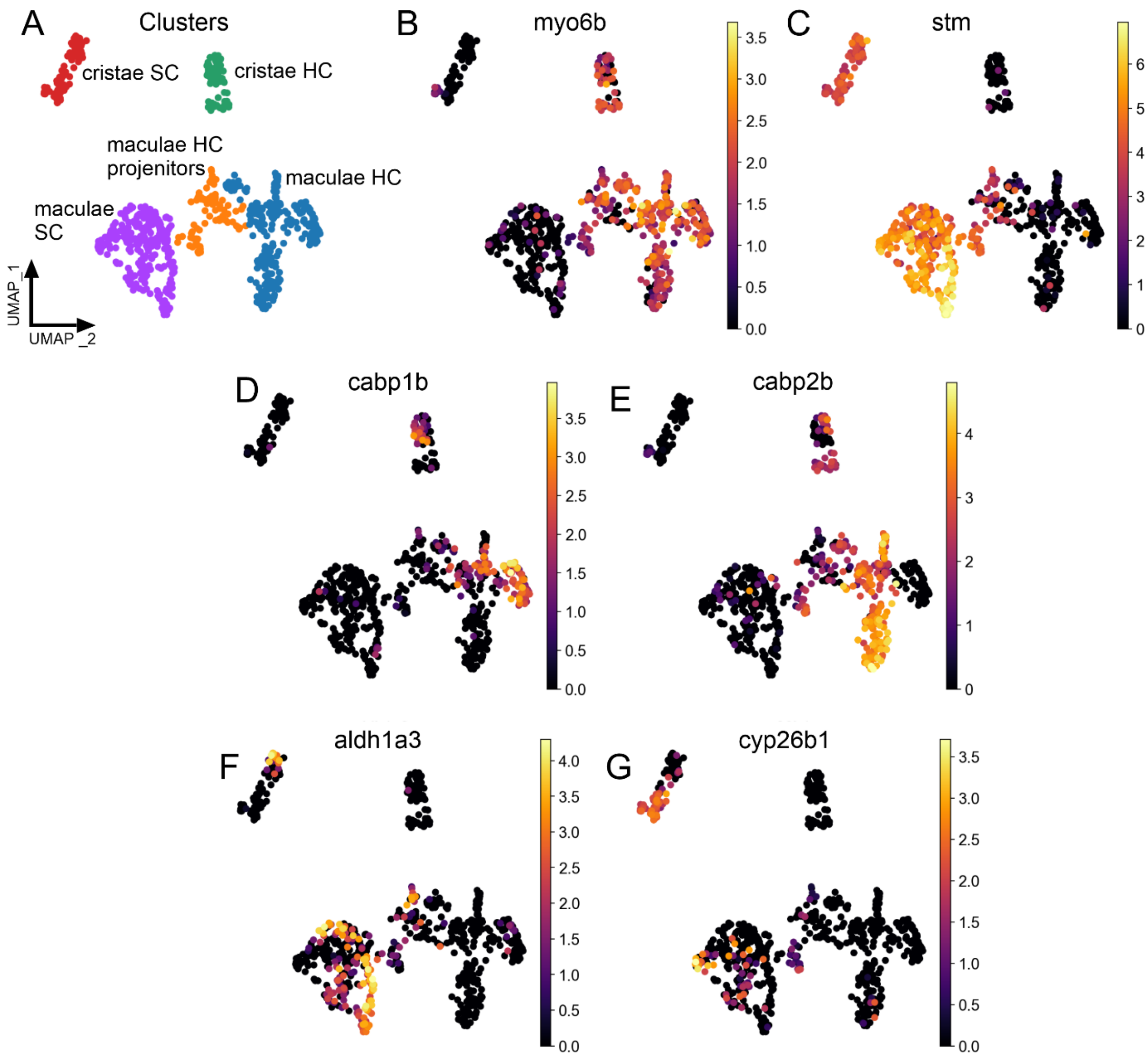

Fig. S1. Single cell RNA sequencing data of larval zebrafish inner ear hair cells and support cells.

(A) UMAP projection of cell grouped by cell type using scRNA seq data from Daniocell. HC: hair cells, SC: support cells.

(B-C) Hair cells were identified using known marker (B) *myo6b* and support cells by (C) *stm*.

(D-E) Hair cells in the maculae can be differentially identified by (D) *cabp1b* or (E) *capb2b*.

(G-H) Supporting cells show differential expression of retinoic acid synthesizing (G) or degrading (H) enzyme RNA.

Development • Supplementary information

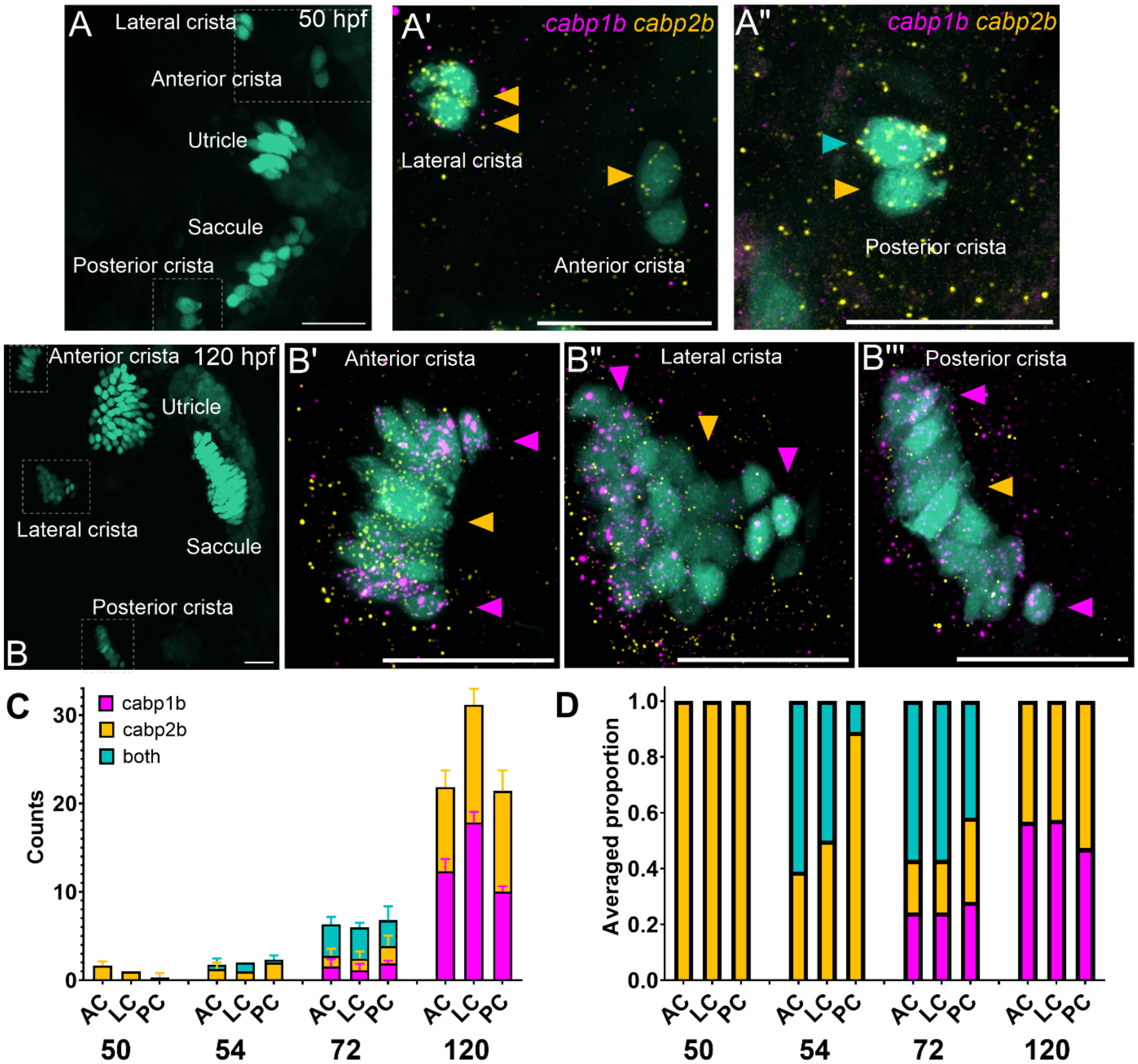

Fig. S2. Identification of cristae hair cell types during larval development.

A-B) Maximum projections of dorsal views of the inner ear with HCR FISH probing for *cabp1b* and *cabp2b* in the cristae of *Tg(myo6b:GFP)* at (A) 50 and (B) 120 hpf. Scale bar = 20 µm. Box plots show mean±s.d.

C-D) Hair cells are added in the first 5 days in the cristae at different rates and the relative proportions of hair cell subtypes change during larval development (n = 3 fish (50 hpf); 3 (54); 9 (72); 6 (120).

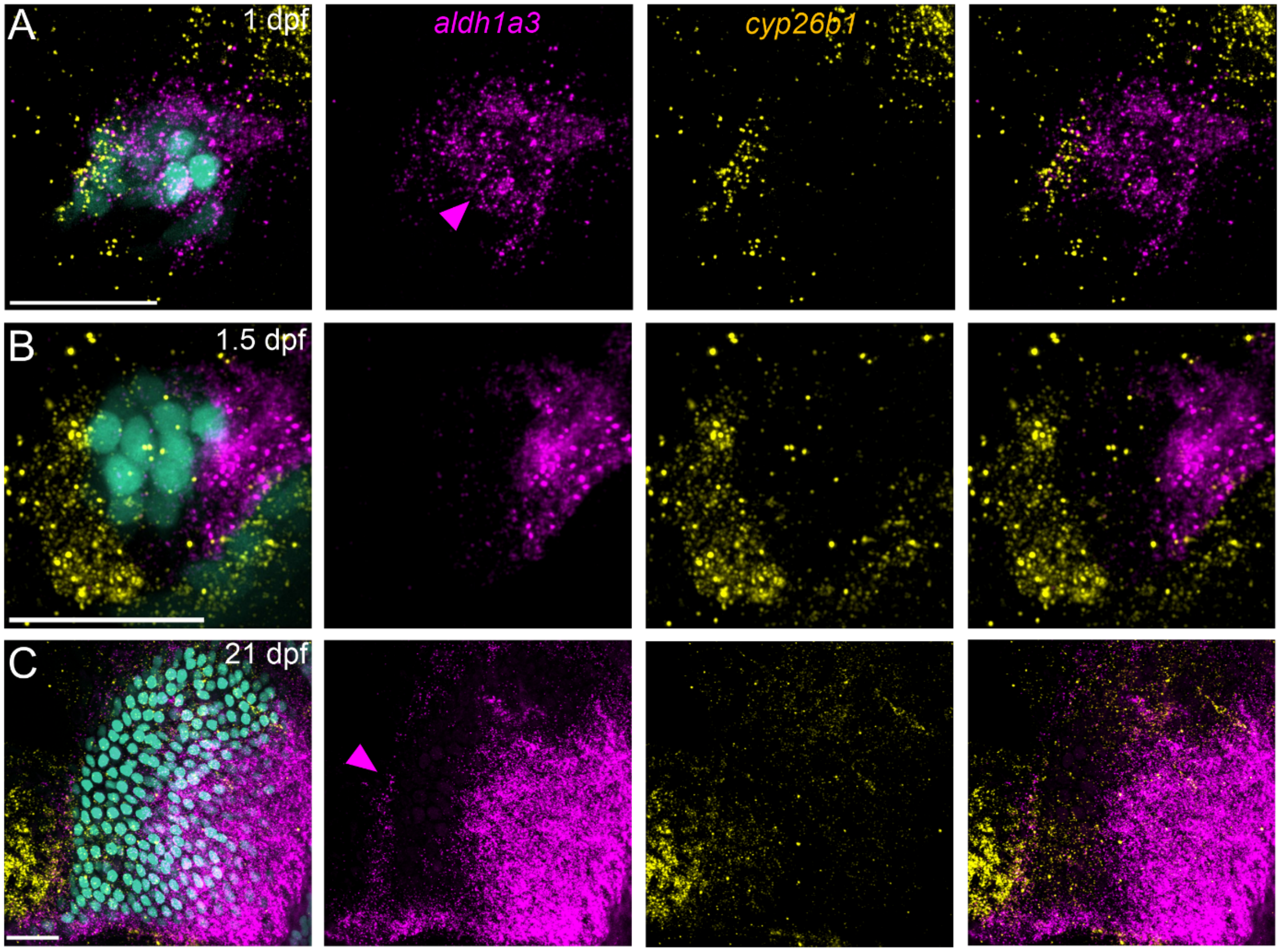

Fig. S3. Patterning of RA enzymes changes with development

A) Maximum projection of a *Tg(myo6b:GFP)* utricle at 1 dpf with probing for *aldh1a3* (magenta) and *cyp26b1* (yellow) shows that very early hair cells are surrounded by *aldh1a3* (magenta arrowhead). Scale bar = 20 μm.

B) At 1.5 dpf (36 hpf), *aldh1a3* has polarized medially as *cyp26b1* expression develops laterally.

C) By 21 dpf, *aldh1a3* expression begins to develop laterally to *cyp26b1* (magenta arrowhead) as the lateral extrastriola begins to form as exemplified here in a *Tg(myo6b:nlsEos)* utricle.

Development • Supplementary information

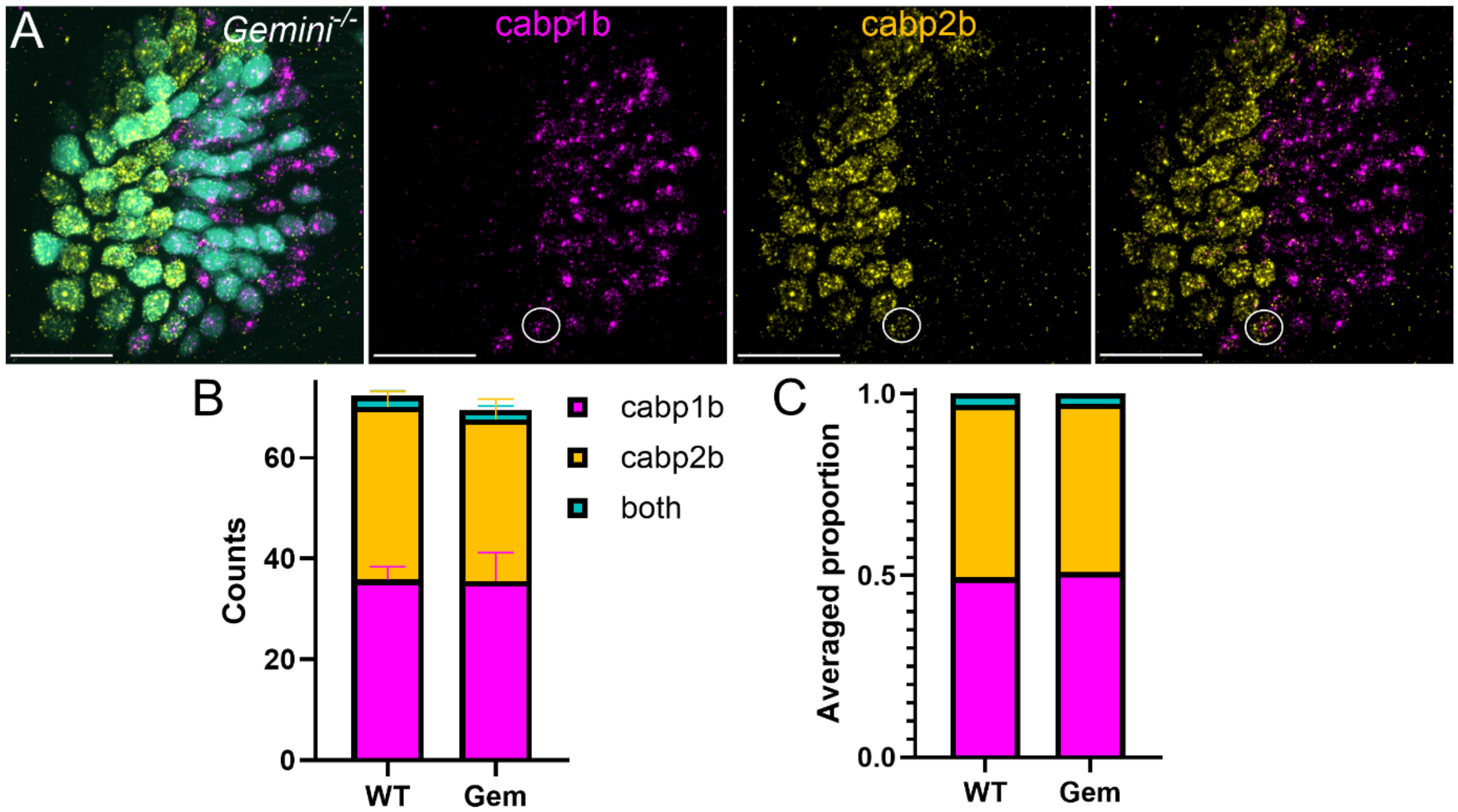

Fig. S4. *Gemini* mutant fish have normal utricular patterning.

A) Maximum projection of *Tg(myo6b:GFP)* utricles in *gemini* mutant fish with HCR FISH probing for *cabp1b* and *cabp2b*. White circle indicates example intermediate (double- labeled) hair cell present at 5 dpf. Scale bar = 20 μm.

B-C) *Gemini* mutants ("Gem") (n = 18 ears across 13 fish) have a similar number of hair cells and similar proportions of striolar/extrastriolar/intermediate hair cells relative to wildtypes (n = 12 ears across 8 fish) at 5 dpf. Box plots show mean ± s.d. in B and mean in C.

Development • Supplementary information

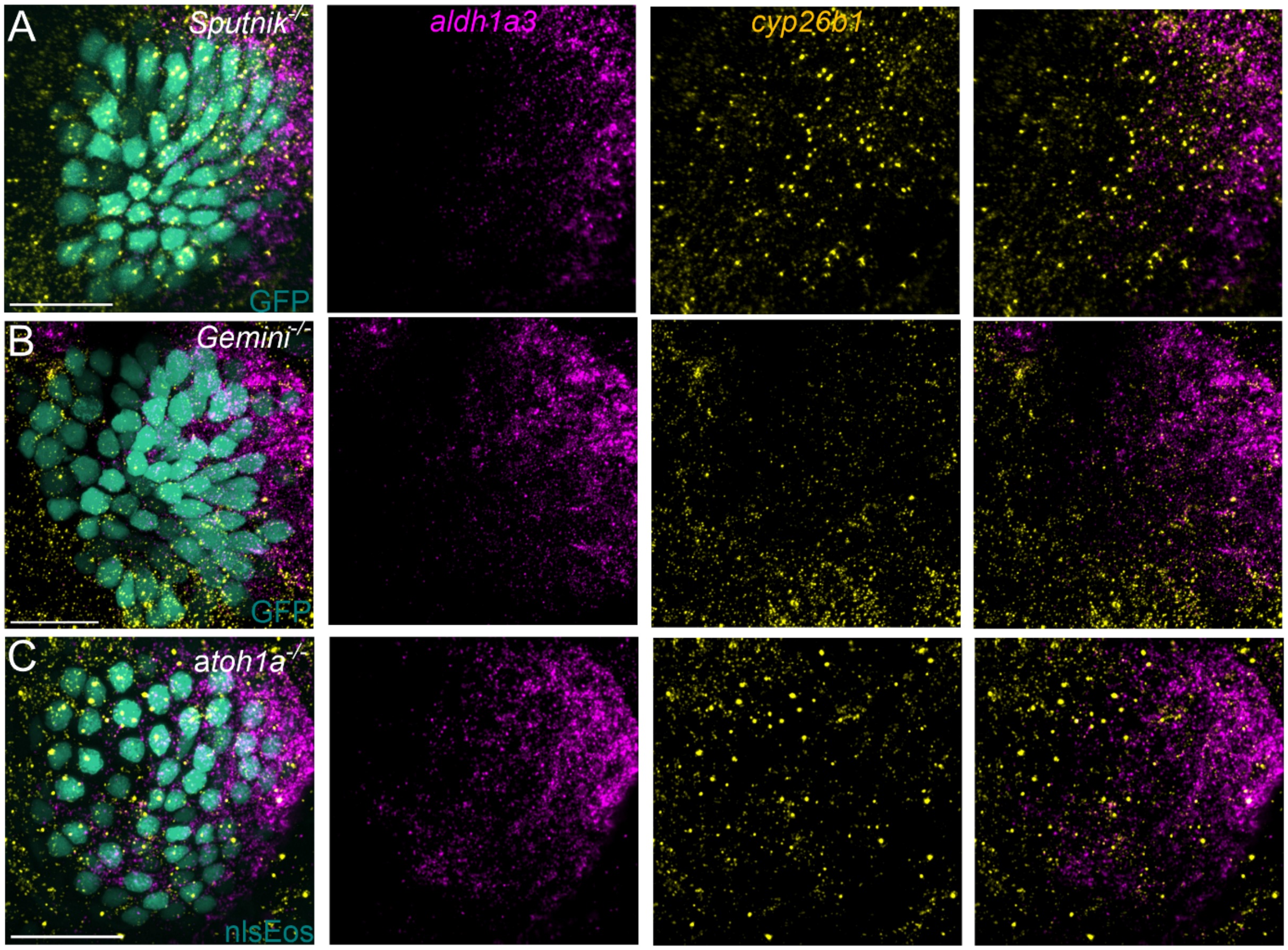

Fig. S5. Fish with hair cell mutations exhibit normal RA enzyme patterning.

Maximum projections of 5 dpf *Tg(myo6b:GFP)* or *Tg(myo6b:nlsEos)* utricles. Scale bar = 20 μm. HCR FISH probing for *aldh1a3* (magenta) and *cyp26b1* (yellow) shows complementary patterning of retinoic acid (RA) enzyme in A) *sputnik*, B) *gemini*, and C) *atoh1a* mutants are comparable to wildtype.

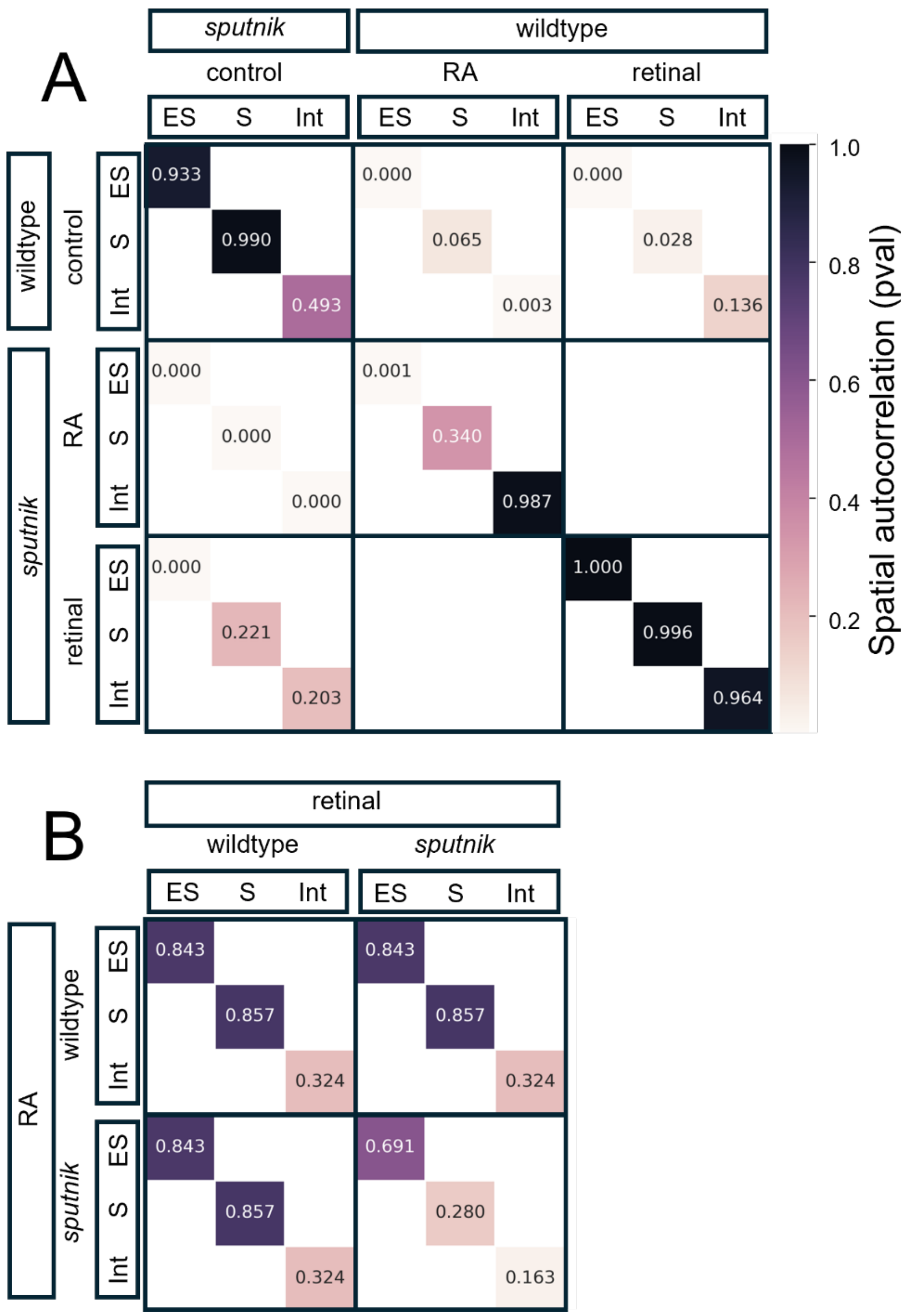

Fig. S6. Spatial autocorrelation analysis confirms differences in zonal patterning across conditions.

Heatmap of spatial autocorrelation between indicated pairs. To determine overlap, nearest neighbor analysis was performed (k=7). Probabilities (pval) were generated by bootstrap analysis.

Development • Supplementary information

Supplemental Methods

Example notebook for expression analysis in the zebrafish utricle

This notebook takes masks generated using Stardist or CellPose, and edited with Napari. The segmented masks can then be used to analyze fluorescent signals. We use the following workflow:

- Import image and masks, correct masks.
- Calculate intensity of HCR signals for each mask, and set cutoffs using intensity histogram and Jenks Natural Breaks.
- Plot centroids of cell masks using polar coordinates.
- Rotate centroids to standardize on the major axis of the utricle by first fitting an ellipse to the concave hull.
- Normaize distances by expanding the convex hull to a circle.

Imports

In [1]:
```
import napari
import numpy as np
import pandas as pd
import skimage as ski
import matplotlib.pyplot as plt
import cv2
import concavity
from matplotlib.colors import ListedColormap
from csbdeep.utils import normalize
from tqdm import tqdm
from tifffile import imread, imwrite
from stardist.data import test_image_nuclei_2d
from stardist.plot import render_label
from stardist import matching as m
from jenkspy import JenksNaturalBreaks
from skimage.morphology import convex_hull_image
from scipy.spatial import ConvexHull, convex_hull_plot_2d
import os
```

Functions

Function to convert cartesian coordinates to polar coordinates

In [3]:
```
def cartesian_to_polar(x,y):
    """
    Converts an array of Cartesian coordinates (x, y) to polar coordinates (
```

```
    Args:
        x,y: A numpy array of shape (N, 2), where N is the number of points.
            Each row represents the cartesian coordinates (x,y) of a point.

    Returns:
        A numpy array of shape (N, 2), where each row represents
        the polar coordinates (r, theta) of the corresponding point.
    """
    r = np.sqrt(x**2 + y**2)
    theta = np.arctan2(y, x)
    return r, theta
```

Function to re-center points on group centroid

In [4]:
```
def recenter_points(points):
    """
    Recenter an array of points such that the centroid is at the origin.

    Args:
        points: dataframe of x,y
        where each row represents a point (x, y).

    Returns:
        dataframe of shape (N, 2),
        where each row represents represents
        the cartesian coordinates (x,y) of the corresponding re-centered poi
    """
    arr = points.to_numpy()
    centroid = np.mean(arr, axis=0)
    recentered_points = arr - centroid
    column_names = ['recentered_x', 'recentered_y']
    df = pd.DataFrame(recentered_points, columns=column_names)
    return df
```

Import and display image

In [5]:
```
print("Current working directory:", os.getcwd())
```

```
Current working directory: c:\Users\baeza\UW\🐟Raible_Lab - Selina\StarDist
```

In [ ]:
```
os.chdir("path/to/your/directory")  # Change to your desired directory
```

In [ ]:
```
# Load image

imgs = imread('utricle.tif')
print(imgs.shape)
```

```
(3, 1244, 1244)
```

In [ ]:
```
# Plot raw image as separate channels; correct for channel order of your ima

# Far red
plt.subplot(1,3,1)
plt.imshow(imgs[0,:,:],cmap="gray")
plt.axis("off")
```

```
plt.title("channel 0")

# Red
plt.subplot(1,3,2)
plt.imshow(imgs[1,:,:],cmap="gray")
plt.axis("off")
plt.title("channel 1")

# Green
plt.subplot(1,3,3)
plt.imshow(imgs[2,:,:],cmap="gray")
plt.axis("off")
plt.title("channel 2")
```

Out[ ]: Text(0.5, 1.0, 'channel 2')

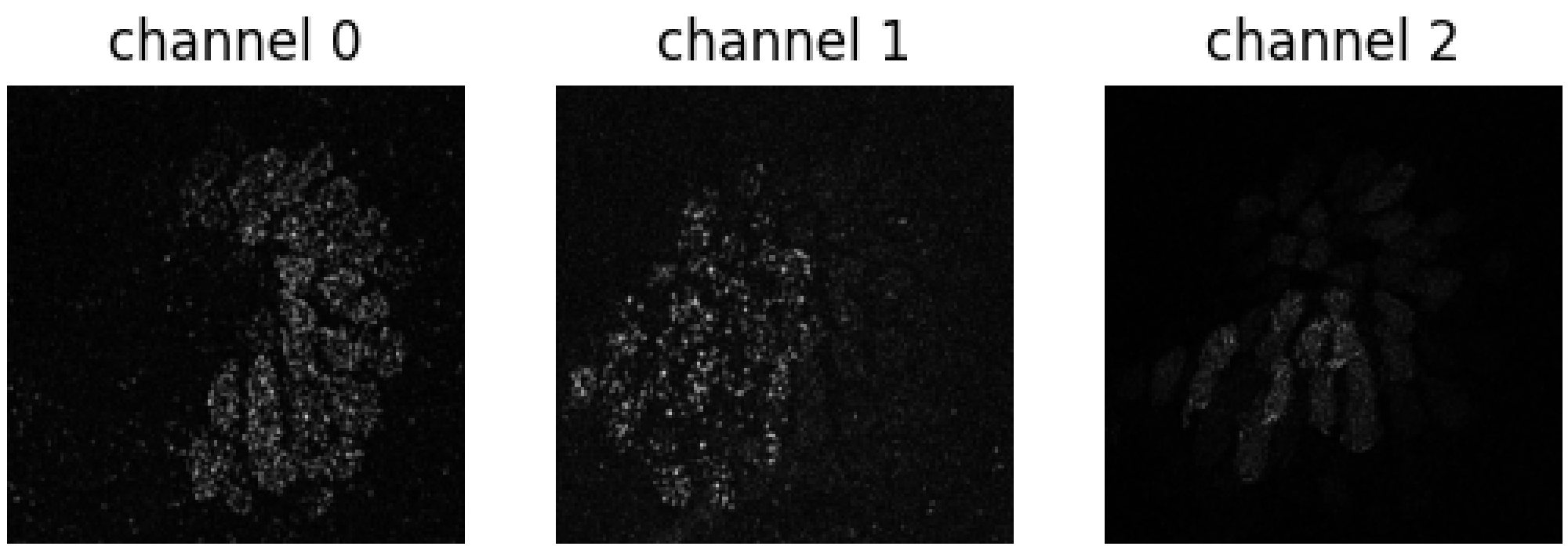

Use Napari to touch up label masks

In [ ]:
```
# Load masks here:
img = imgs[2,:,:]
labels = imread('masks.tif')
```

In [ ]:
```
# Open, view, and edit masks with Napari

viewer = napari.Viewer()
new_layer = viewer.add_image(img, name='green')
new_layer = viewer.add_image(imgs[1,:,:], name='red')
new_layer = viewer.add_image(imgs[0,:,:], name='far red')
labels_layer = viewer.add_labels(labels, name='mask')
#labels_layer = viewer.add_labels(labels_f, name='filtered_mask')
```

In [15]:
```
# Double check labels
print (len(np.unique(labels)))
```

64

In [16]:
```
# Plot masks over green channel image

plt.imshow(render_label(labels, img=img))
plt.axis("off")
#plt.title("masks")
```

Out[16]: (-0.5, 1243.5, 1243.5, -0.5)

Development • Supplementary information

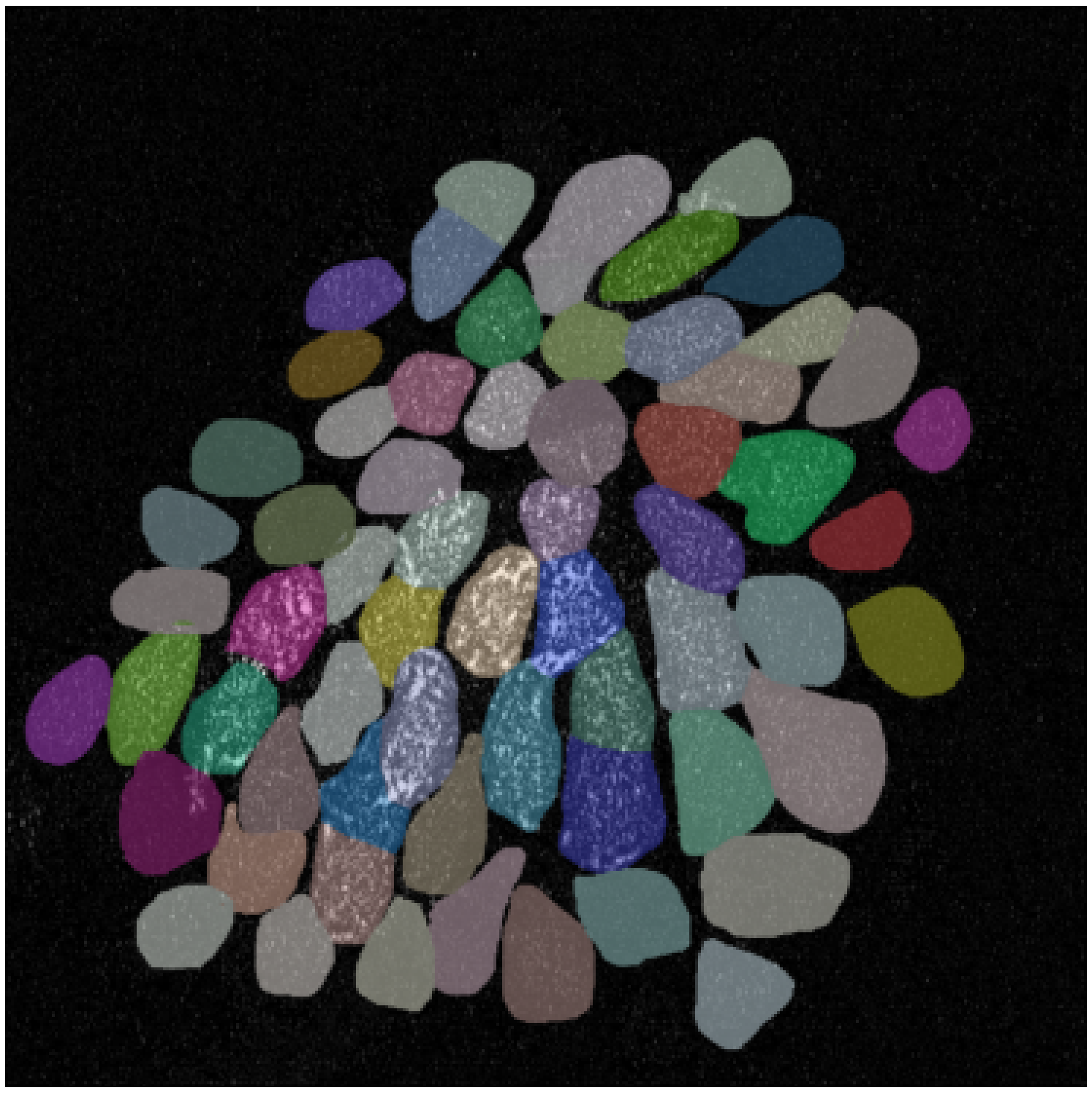

Save mask file

In [ ]:
```
# Make sure to use a different name for each mask file
imwrite('corrected_mask.tif', labels)
```

Get fluorescent values and mask centroids, generate prop table

In [ ]:
```
# Reload images and mask files.  You can also start from here for analysis.

imgs = imread('utricle.tif') #
image_g = imgs[2,:,:]
image_r = imgs[1,:,:]
image_fr = imgs[0,:,:]
label_image = imread('masks.tif') #labels
print (len(np.unique(label_image)))
```

64

create data table ('prop' table)

In [29]:
```
# function to calculate integrated density over each labeled segment in a ma
def integrated_density(regionmask, intensity_image):
    return np.sum(intensity_image)

#get region props for green channel (all nuclei)
props_g = pd.DataFrame(
        ski.measure.regionprops_table(
                label_image, intensity_image=image_g,
                properties=['label', 'intensity_mean', 'centroid'],
                )
```

```
        )
props_g = props_g.rename(columns={'intensity_mean': 'mean_g'})

#get region props for red channel
#ADD integrated_density if having another HCR marker in red channel
props_r = pd.DataFrame(
        ski.measure.regionprops_table(
                label_image, intensity_image=image_r,
                properties=['intensity_mean'],
                extra_properties=(integrated_density,)
                )
        )
props_r = props_r.rename(columns={'intensity_mean': 'mean_r','integrated_der

#get region props for far-red channel
props_fr = pd.DataFrame(
        ski.measure.regionprops_table(
                label_image, intensity_image=image_fr,
                properties=['intensity_mean'],
                extra_properties=(integrated_density,)
                )
        )
props_fr = props_fr.rename(columns={'intensity_mean': 'mean_fr','integrated_

#combine regionprops dataframes
merged_props = pd.concat([props_g, props_r, props_fr], axis=1)
#merged_props['r_g_ratio'] = merged_props['mean_r']/merged_props['mean_g']
```

Alternative way to calculate distances between points and center. Using Python with complex numbers

In [ ]:
```
#Recenter centroid coordinates to new origin (group centroid)
df = recenter_points(merged_props[['centroid-1','centroid-0']])  #regionprop
merged_props = pd.concat([merged_props, df], axis=1)
merged_props['recentered_y'] = -merged_props['recentered_y'] #correct for or
merged_props['rad'], merged_props['theta'] = cartesian_to_polar(merged_props

#use complex numbers to calculate euclidean distance
#first calculate center
center_x  = merged_props['centroid-1'].mean()
center_y  = merged_props['centroid-0'].mean()
center_xy = center_x + center_y * 1j

merged_props['xy'] = merged_props['centroid-1'] + merged_props['centroid-0']
merged_props['distance'] = (merged_props['xy'] - center_xy).abs()
```

Plot histograms and set cutoffs

Plot histogram of red channel

In [31]:
```
merged_props.hist(column='mean_r', bins=50)
```

Out[31]:
```
array([[<Axes: title={'center': 'mean_r'}>]], dtype=object)
```

Development • Supplementary information

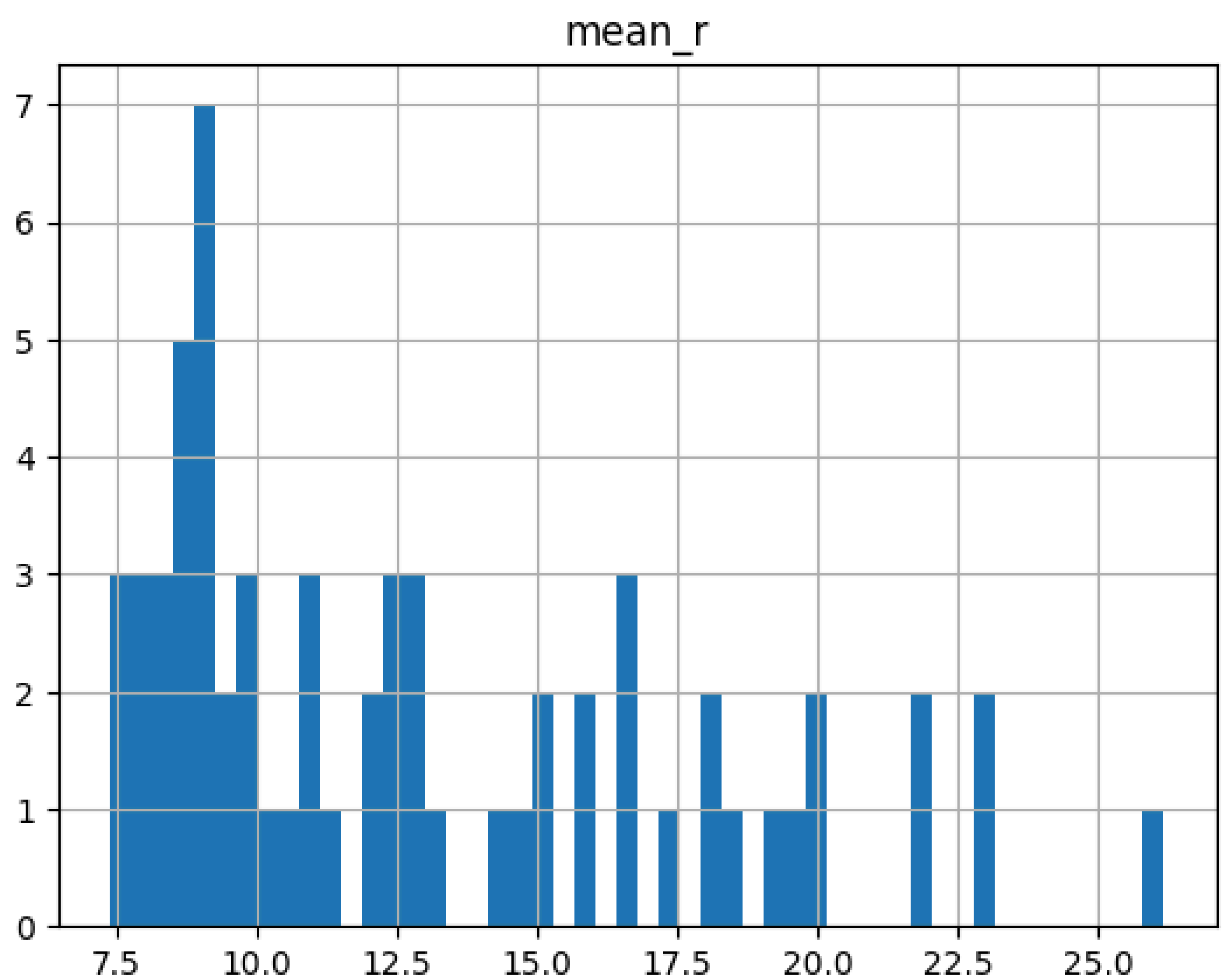

In [32]:
```
jnb = JenksNaturalBreaks(5) # Asking for 5 clusters - I find this separates
jnb.fit(merged_props['mean_r']) # Create the clusters
r_cutoff = jnb.inner_breaks_[1] #np.float64(310)  #
print(jnb.inner_breaks_)
```

```
[10.013262854396924, 13.289835164835164, 17.33953153153153, 19.9639477242000
9]
```

Plot histogram of far red signal

In [33]:
```
merged_props.hist(column='mean_fr', bins=50)
```

Out[33]:
```
array([[<Axes: title={'center': 'mean_fr'}>]], dtype=object)
```

Development • Supplementary information

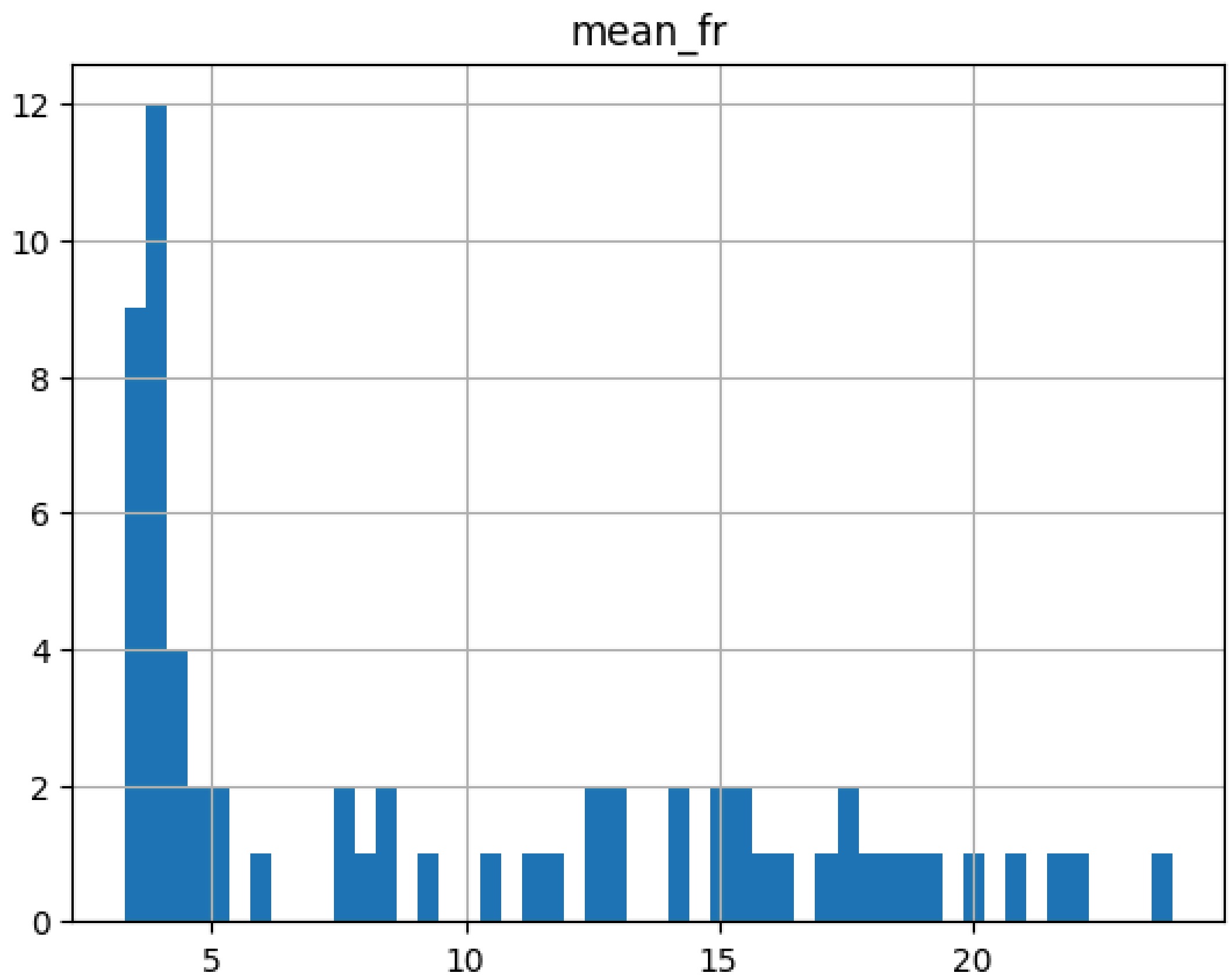

In [34]:
```
jnb = JenksNaturalBreaks(5) # Asking for 5 clusters - I find this separates
jnb.fit(merged_props['mean_fr']) # Create the clusters
c_cutoff = jnb.inner_breaks_[1]
print(jnb.inner_breaks_)
```

```
[5.833707461614439, 9.325778546712803, 14.23820143884892, 19.12125902992776]
```

Label mask image with values

Functions

In [35]:
```
# Convert labels to float
merged_props = merged_props.astype({'label': 'float64'})
# Create dictionaries
dist_dict = dict(zip(merged_props['label'], merged_props['rad']))


def label_mask_image (input_mask, value_dict):
    """
    Generate a labeled mask image using a dictionary of labels and values.
    """
    input_mask = input_mask.astype(np.float64)
    labels = np.unique(input_mask)
    for label in labels:
        if label == 0.0: #skip background
            continue
        input_mask[input_mask == label] = value_dict[label]
    return input_mask
```

Development • Supplementary information

In [37]:
```
def filter_col (df, col_to_filter, cutoff, selection= "", colname = "" ):
    """
    Generate a Boolean mask for a dataframe column, selecting either
    <= ('under') or > ('over') cutoff.
    Return df with Boolean values in column named colname.
    """
    conditions = [ (df[col_to_filter]<=cutoff), (df[col_to_filter]>cutoff)]
    if selection == "under":
        values = [1,0]
    else:
        values = [0,1]
    df[colname] = np.select(conditions, values)
    return df
```

Select red cells

In [38]:
```
# Select red cells based on cutoff

filter_val = r_cutoff
merged_props = filter_col(merged_props, 'mean_r', filter_val, selection = "c
```

In [40]:
```
## Plot red cells

#create dict from boolean mask, adding 1 for colormap indexing
str_dict = dict(zip(merged_props['label'], (merged_props['red']+1)))
#generate labeled mask
cg_ratio_mask_2 = label_mask_image(label_image, str_dict)

count_ones = (merged_props["red"] == 1).sum()
print("Number of red cells:", count_ones)

# Define colors for each value
colors = {0:'black', 1: '#C8C8C8BA', 2:'#FF00FF'}

# Create a colormap from the defined colors
cmap_cg = ListedColormap([colors[i] for i in np.unique(cg_ratio_mask_2)])

#print (len(np.unique(cg_ratio_mask_2)))

# Plot the data using the custom colormap
plt.imshow(cg_ratio_mask_2, cmap=cmap_cg, interpolation='nearest')

plt.axis("off")
#plt.title("cabp1b+")
```

```
Number of red cells: 34
```

Out[40]:
```
(-0.5, 1243.5, 1243.5, -0.5)
```

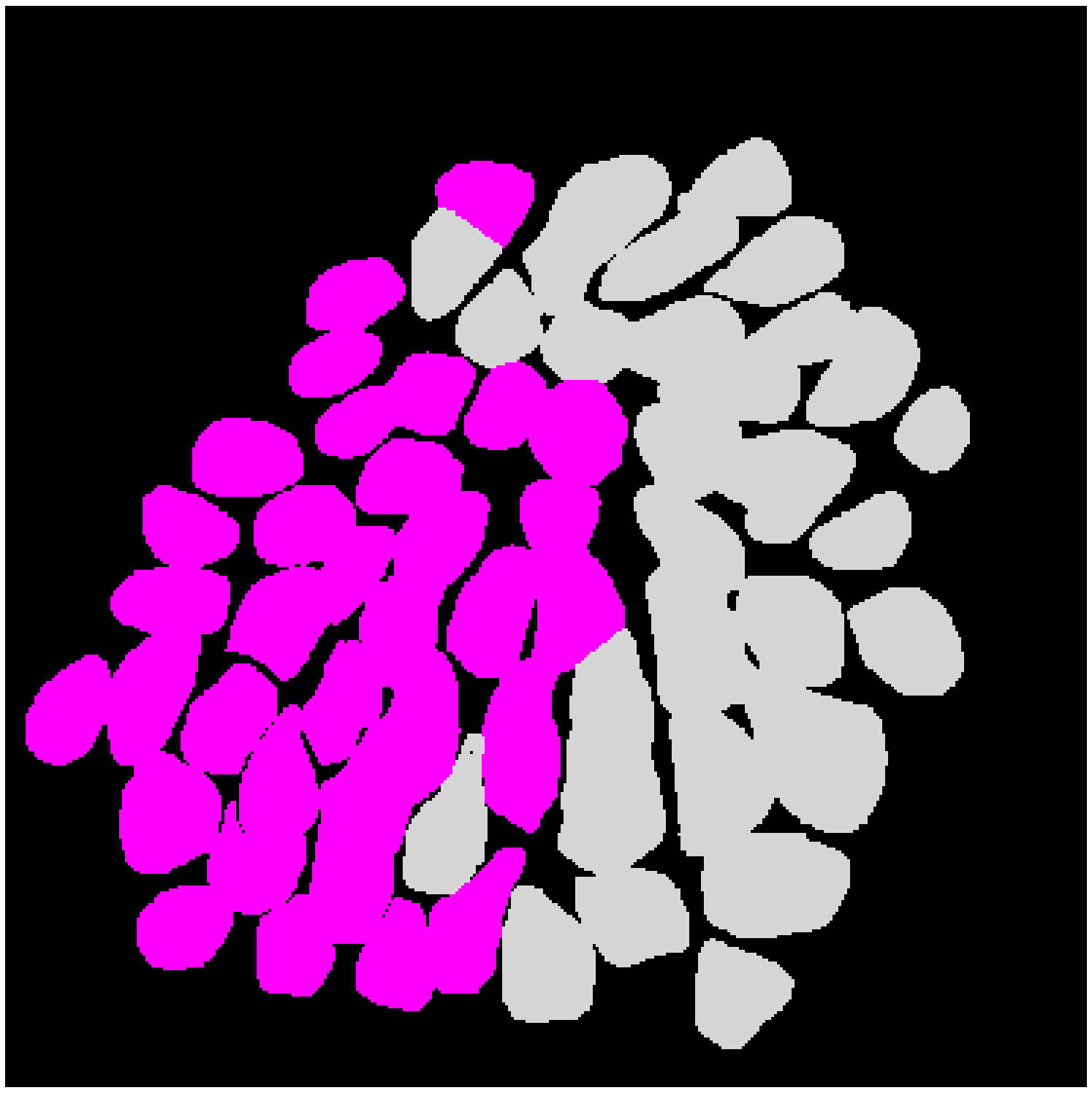

Select far red cells

In [ ]:
```
# Select far red cells based on cutoff

filter_val = c_cutoff
merged_props = filter_col(merged_props, 'mean_fr', filter_val, selection = '
```

In [41]:
```
## Plot far red cells
#create dict from boolean mask, adding 1 for colormap indexing
fr_dict = dict(zip(merged_props['label'], (merged_props['far red']+1)))
#generate labeled mask
fr_ratio_mask_2 = label_mask_image(label_image, fr_dict)

count_ones = (merged_props["far red"] == 1).sum()
print("Number of far red cells:", count_ones)

# Define colors for each value
colors = {0:'black', 1:'#C8C8C8BA', 2:'#FFFF14'}

# Create a colormap from the defined colors
cmap_cg = ListedColormap([colors[i] for i in np.unique(fr_ratio_mask_2)])

# Plot the data using the custom colormap
plt.imshow(fr_ratio_mask_2, cmap=cmap_cg, interpolation='nearest')

plt.axis("off")
#plt.title("cabp2b+")
```

```
Number of far red cells: 33
```

Out[41]:
```
(-0.5, 1243.5, 1243.5, -0.5)
```

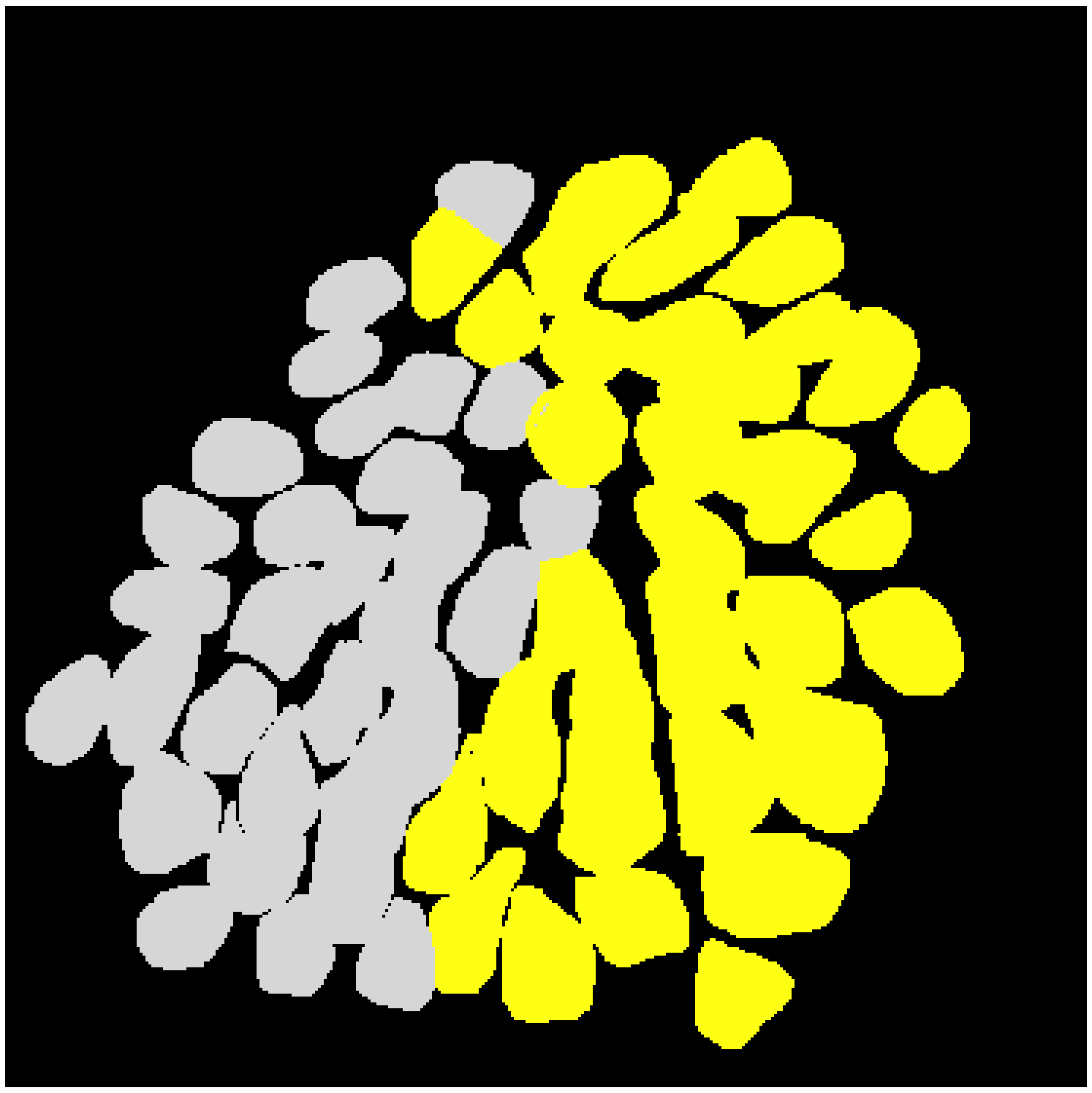

Open all plots as masks in Napari

In [ ]:
```
viewer = napari.Viewer()
new_layer = viewer.add_image(imgs[2,:,:], name='green')
new_layer = viewer.add_image(imgs[1,:,:], name='red')
new_layer = viewer.add_image(imgs[0,:,:], name='far red')
labels_layer = viewer.add_labels(cg_ratio_mask_2.astype(np.int32), name='cg_
labels_layer = viewer.add_labels(fr_ratio_mask_2.astype(np.int32), name='fr_
labels_layer = viewer.add_labels(label_image, name='mask')
```

Select double positive red + far red cells

In [42]:
```
double_labeled = (merged_props["red"] == 1) & (merged_props["far red"] == 1)
count_double_labeled = double_labeled.sum()
print("double labeled cells:", count_double_labeled)
```

```
double labeled cells: 4
```

In [43]:
```
# Plot double labeled
fr_str_dict = dict(zip(merged_props['label'], (merged_props['red']*(merged_p
fr_str_mask = label_mask_image(label_image, fr_str_dict)

# Define colors for each value
colors = {0:'black', 1:'black' , 2: "#14FFC0"}

# Create a colormap from the defined colors
cmap_estr = ListedColormap([colors[i] for i in np.unique(fr_str_mask)])

# Plot the data using the custom colormap
plt.imshow(fr_str_mask, cmap=cmap_estr, interpolation='nearest')
```

```
plt.axis("off")
#plt.title("double labeled")
```

Out[43]: (-0.5, 1243.5, 1243.5, -0.5)

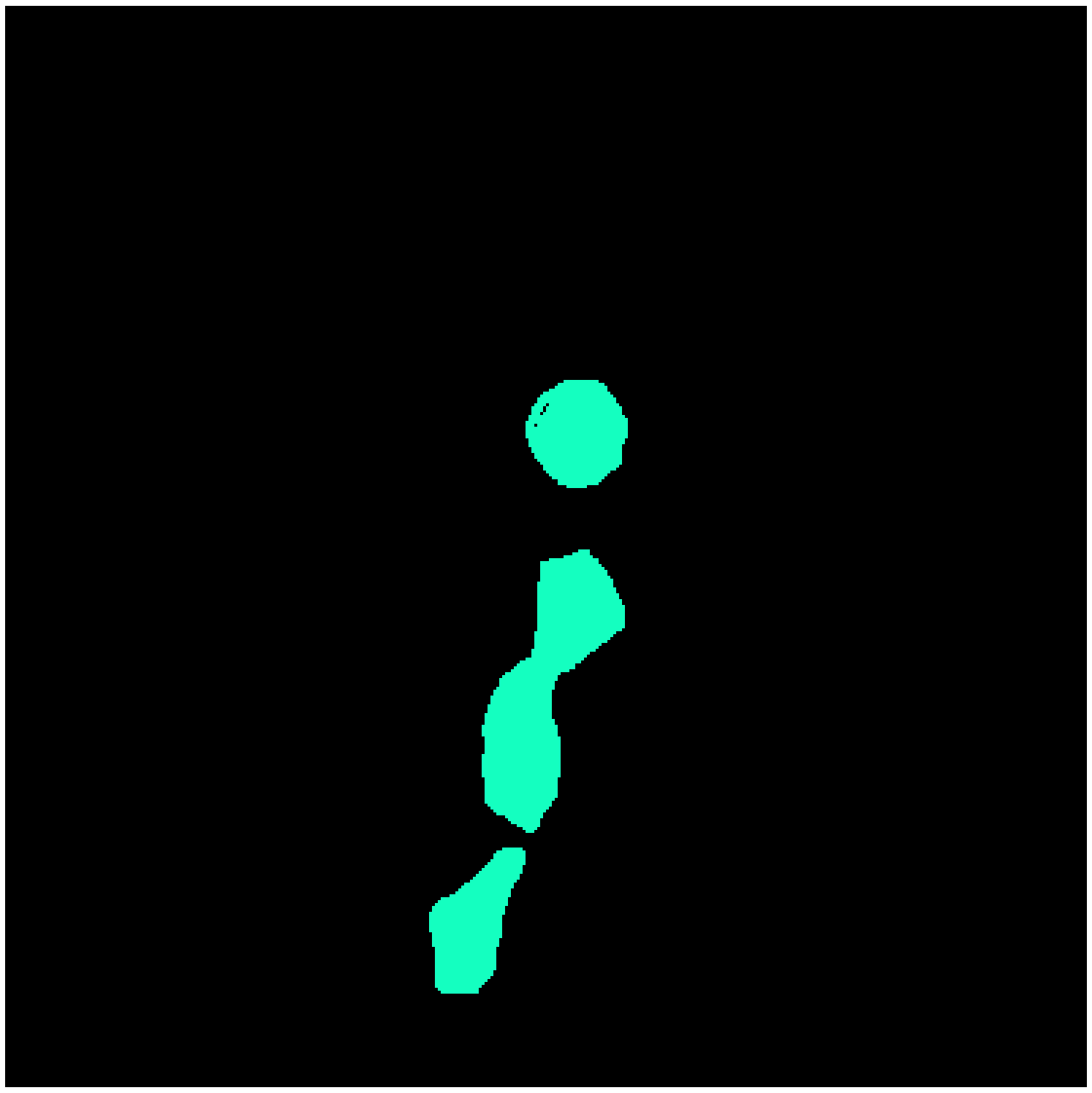

Draw convex hull

In [44]:
```
one_list= [1]*len(merged_props['label'])
one_dict = dict(zip(merged_props['label'], one_list))
one_mask = label_mask_image(label_image, one_dict)
```

In [45]:
```
# Define colors for each value
colors = {0:'black', 1: 'white'}

# Create a colormap from the defined colors
cmap_one = ListedColormap([colors[i] for i in np.unique(one_mask)])

# Plot the data using the custom colormap
plt.imshow(one_mask, cmap=cmap_one, interpolation='nearest')

plt.axis("off")
plt.title("input image")
```

Out[45]: Text(0.5, 1.0, 'input image')

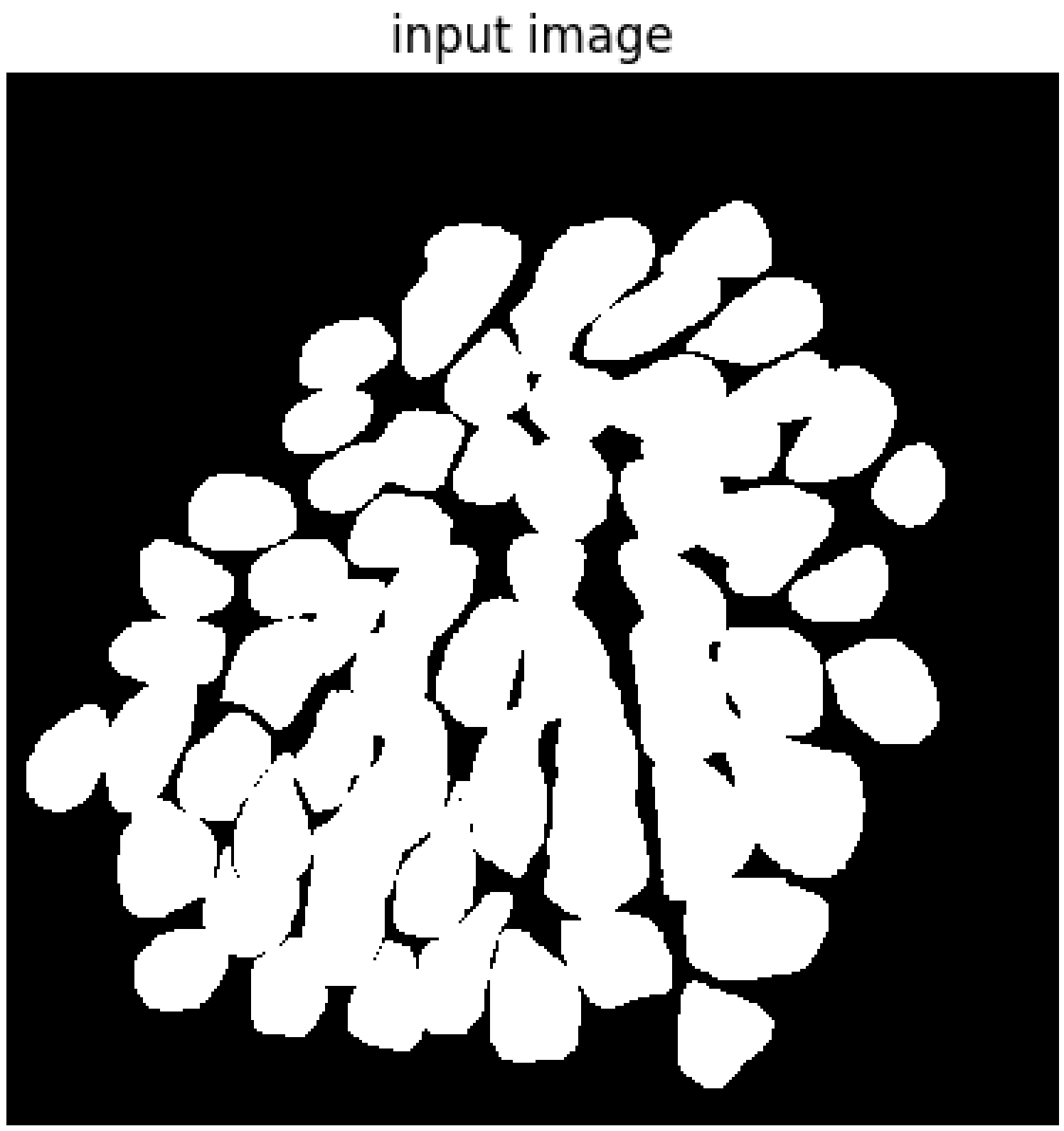

In [46]:
```
points = merged_props[['centroid-1','centroid-0']].to_numpy() # centroids ir
hull = ConvexHull(points)
simplices = points[hull.simplices]
print (len(simplices))
```

10

In [47]:
```
plt.plot(points[:,0], points[:,1], 'o')
for simplex in hull.simplices:
    plt.plot(points[simplex, 0], points[simplex, 1], 'w--')
plt.imshow(one_mask, cmap=cmap_one, interpolation='nearest')
plt.axis("off")
plt.title("input image")
```

Out[47]: Text(0.5, 1.0, 'input image')

Development • Supplementary information

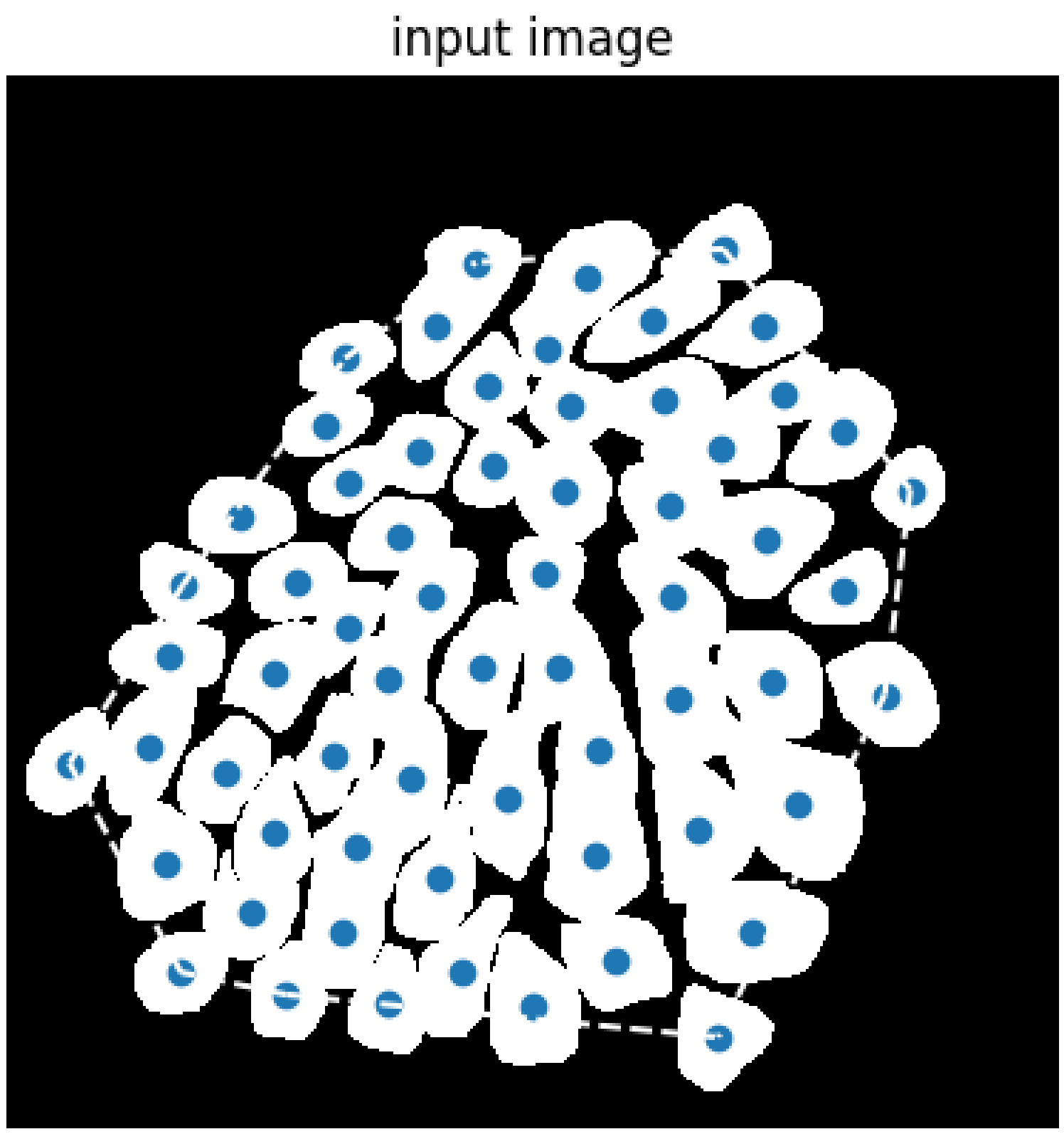

In [48]:
```
plt.plot(merged_props['recentered_x'],merged_props['recentered_y'], 'o')
#plt.imshow(one_mask, cmap=cmap_one, interpolation='nearest')
#plt.axis("off")
plt.gca().set_aspect('equal')
plt.title("input image")
```

Out[48]:
```
Text(0.5, 1.0, 'input image')
```

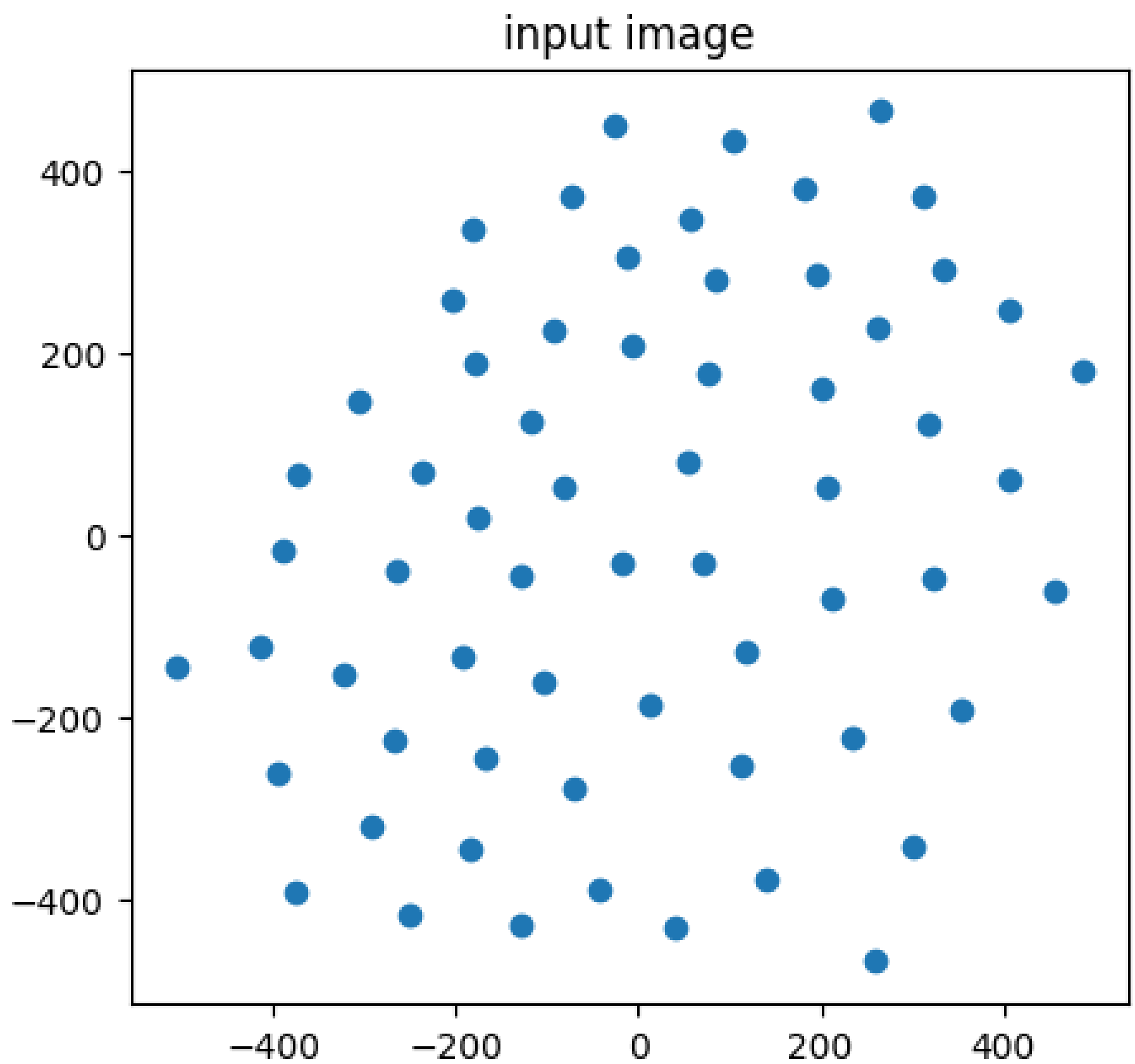

Save

```
In [ ]: merged_props.to_csv('merged_props.csv', index=False)
```

Create Polar Plots

Rotate points using fitted ellipse and expand to normalized polar plot

Use concavity method to find concave hull with nearest neighbors

Imports

```
In [49]: import numpy as np
         import pandas as pd
         from numpy.linalg import eig, inv
         from shapely.geometry import MultiPoint, Point
         from shapely import convex_hull
         import geopandas as gpd
         import matplotlib.pyplot as plt
         import concavity
         import os
         import glob
```

Development • Supplementary information

Transformation functions

In [50]:
```
def recenter_points(points):
    """
    Recenter an array of points such that the centroid is at the origin.

    Args:
        points (numpy.ndarray): An array of points,
        where each row represents a point (x, y).

    Returns:
        numpy.ndarray: A new A numpy array of shape (N, 2),
        where each row represents represents
        the cartesian coordinates (x,y) of the corresponding re-centered poi
    """
    centroid = np.mean(points, axis=0)
    recentered_points = points - centroid
    return recentered_points
```

In [51]:
```
def cartesian_to_polar(xy):
    """
    Converts an array of Cartesian coordinates (x, y) to polar coordinates (

    Args:
        xy: A numpy array of shape (N, 2), where N is the number of points.
            Each row represents the cartesian coordinates (x,y) of a point.

    Returns:
        A numpy array of shape (N, 2), where each row represents
        the polar coordinates (r, theta) of the corresponding point.
    """
    x = xy[:, 0]
    y = xy[:, 1]
    r = np.sqrt(x**2 + y**2)
    theta = np.arctan2(y, x)
    return np.stack((r, theta), axis=-1)
```

In [52]:
```
def polar_to_cartesian(rtheta):
    """
    Converts an array of polar coordinates (r, theta) to cartesian coordinat

    Args:
        rtheta: A numpy array of shape (N, 2), where N is the number of poir
            Each row represents a point in polar coordinates (r, theta).

    Returns:
        A numpy array of shape (N, 2), where each row represents
        the cartesian coordinates (x,y) of the corresponding point.
    """
    r = rtheta[:, 0]
    theta = rtheta[:, 1]
    x = r * np.cos(theta)
```

```
    y = r * np.sin(theta)
    return np.stack((x,y), axis=-1)
```

In [53]:
```
def fit_ellipse(x, y):
    x = x[:, np.newaxis]
    y = y[:, np.newaxis]
    D = np.hstack((x * x, x * y, y * y, x, y, np.ones_like(x)))
    S = np.dot(D.T, D)
    C = np.zeros([6, 6])
    C[0, 2], C[2, 0], C[1, 1] = 2, 2, -1
    U, s, V = np.linalg.svd(np.dot(inv(S), C))
    a = U[:, 0]
    return a

def ellipse_center(a):
    b, c, d, f, g, a = a[1] / 2, a[2], a[3] / 2, a[4] / 2, a[5], a[0]
    num = b * b - a * c
    x0 = (c * d - b * f) / num
    y0 = (a * f - b * d) / num
    return np.array([x0, y0])

def ellipse_angle_of_rotation(a):
    b, c, d, f, g, a = a[1] / 2, a[2], a[3] / 2, a[4] / 2, a[5], a[0]
    return 0.5 * np.arctan((2 * b) / (a - c))

def ellipse_axis_length(a):
    b, c, d, f, g, a = a[1] / 2, a[2], a[3] / 2, a[4] / 2, a[5], a[0]
    up = 2 * (a * f * f + c * d * d + g * b * b - 2 * b * d * f - a * c * g)
    down1 = (b * b - a * c) * ((c - a) * np.sqrt(1 + 4 * b * b / ((a - c) *
    down2 = (b * b - a * c) * ((a - c) * np.sqrt(1 + 4 * b * b / ((a - c) *
    res1 = np.sqrt(up / down1)
    res2 = np.sqrt(up / down2)
    return np.array([res1, res2])
```

In [54]:
```
def c_hull(coords):
    """
    Calculates convex hull around array of points.

    Args:
        coords: x,y coordinate array

    Returns:
        convex hull as Multipoint boundary
    """
    points = list(map(Point, coords))
    multi_point = MultiPoint(points)
    hull = convex_hull(multi_point).boundary
    return hull
```

In [55]:
```
def boundary_points(hull, interval=360):
    """
    Calculates boundary points around convex hull at specific linear interva

    Args:
        hull: Multipoint convex hull
```

Development • Supplementary information

```
        interval: number of intervals
    Returns:
        array of x,y boundary points
    """
    boundary = np.zeros(shape=(interval,2))
    for i in range(interval):
        xpoint = hull.interpolate((i/interval), normalized=True)
        boundary[i,0] = xpoint.x
        boundary[i,1] = xpoint.y
    return boundary
```

In [56]:
```
def make_factor_dict(polar_boundary):
    """
    Calculates factors to reshape points array using convex hull boundary pc
    dictionary of r factors for each theta.

    Args:
        polar_boundary:  array of boundary points in polar coords (r, theta)

    Returns:
        dict of r factors for theta keys
    """
    r_b = polar_boundary[:, 0]
    theta_b =  polar_boundary[:, 1]
    r_max = r_b.max()
    r_factor = r_max/r_b
    factor_dict = dict(zip(theta_b,r_factor))
    return factor_dict
```

In [57]:
```
def closest_val(test_dict, test_key):
    """
    Find value from dictionary for key closest to test key.

    Args:
        test_dict: dictionary to query
        test_key:  query key
    Returns:
        value for key
    """
    keys = np.asarray(list(test_dict.keys()))
    closest = (np.abs(keys - test_key)).argmin()
    val = test_dict[keys[closest]]
    return val
```

Load data

In [ ]:
```
merged_props = pd.read_csv("merged_props.csv")
```

Generate normalized polar plot

In [59]:
```
# Updated data table

x = merged_props['recentered_x']
```

Development • Supplementary information

```
y = merged_props['recentered_y']
coords = merged_props[['recentered_x', 'recentered_y']].to_numpy()

plt.plot(x, y, 'o')
plt.gca().set_aspect('equal')
plt.title("input image")
```

Out[59]: Text(0.5, 1.0, 'input image')

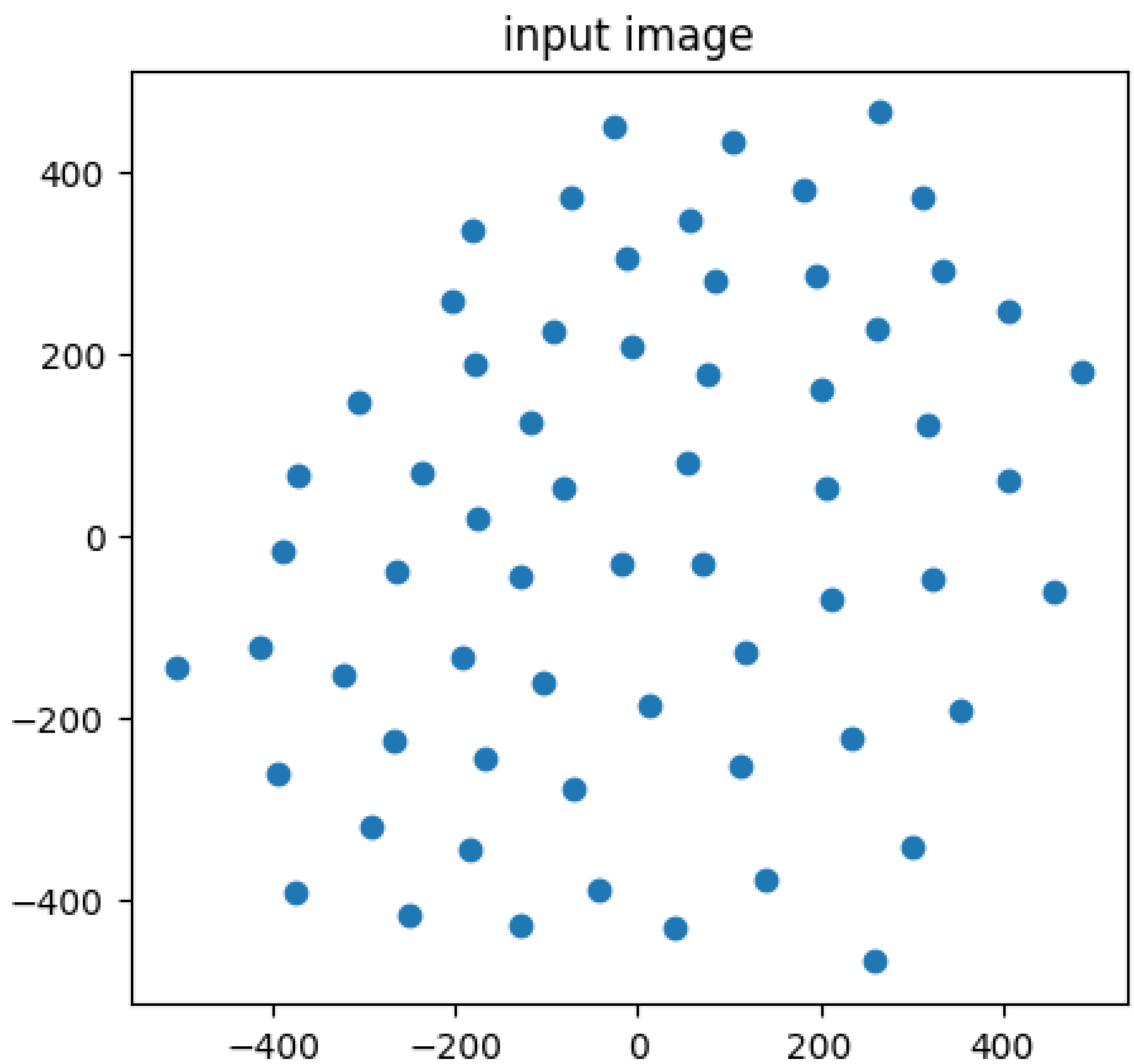

In [60]:
```
# get concave hull with nearest neighbors of 3
ch = concavity.concave_hull(coords, 3)
concavity.plot_concave_hull(coords, ch)
```

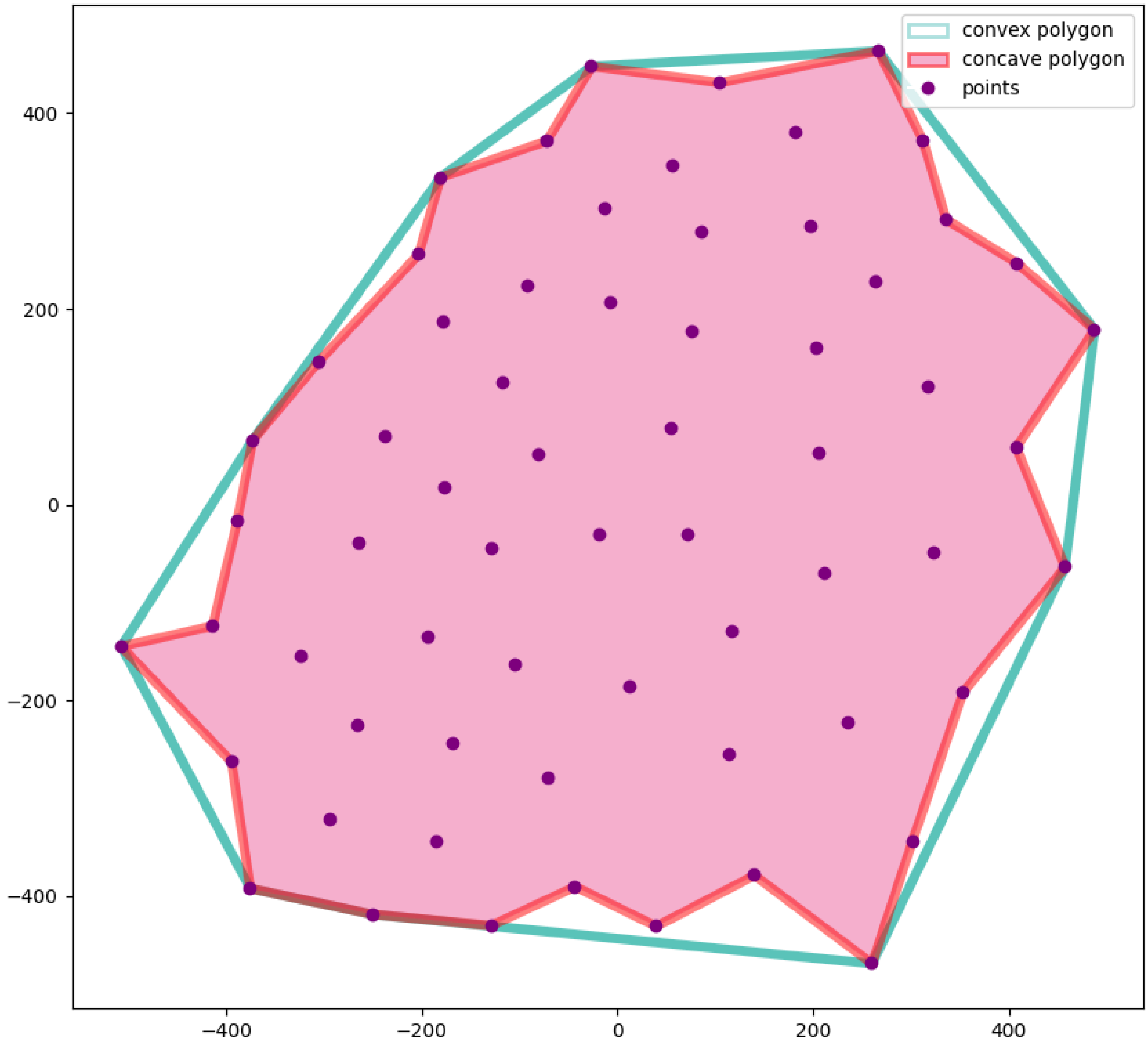


```
In [61]: # Get exterior vertices of convex hull
vertices_array = np.asarray(ch.exterior.coords)
vertices_array_x = vertices_array[:,0]
vertices_array_y = vertices_array[:,1]

# Fit ellipse
coeffs = fit_ellipse(vertices_array_x, vertices_array_y)
center = ellipse_center(coeffs)
angle = ellipse_angle_of_rotation(coeffs)
axis_lengths = ellipse_axis_length(coeffs)

# Plot the results
plt.figure(figsize=(6, 6))
plt.plot(vertices_array_x, vertices_array_y, 'o', label='Data Points')

# Generate points on the fitted ellipse
theta_grid = np.linspace(0, 2*np.pi, 200)
x_ellipse = center[0] + axis_lengths[0] * np.cos(theta_grid) * np.cos(angle)
y_ellipse = center[1] + axis_lengths[0] * np.cos(theta_grid) * np.sin(angle)

plt.plot(x_ellipse, y_ellipse, '-', label='Fitted Ellipse')
plt.plot(*center, 'r*', label='Ellipse Center')
```

```
plt.xlabel('x')
plt.ylabel('y')
plt.title('Ellipse Fitting')
plt.legend()
plt.grid(True)
plt.show()
```

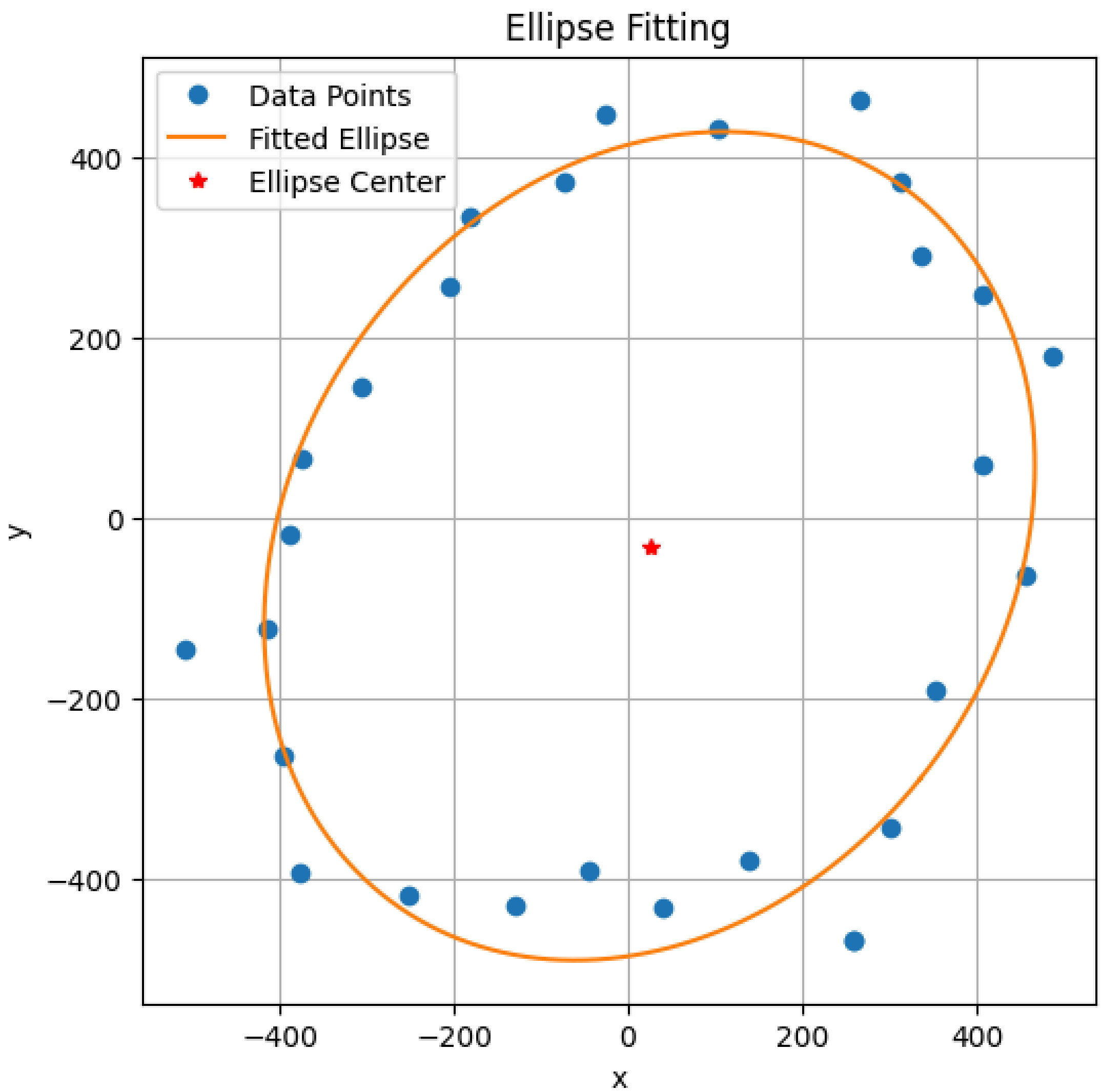

In [62]:
```
# Convert to polar
polar_coords = cartesian_to_polar(coords)
polar_vertices = cartesian_to_polar(vertices_array)

# Rotate by ellipse angle of rotation
corr_polar_coords = polar_coords.copy()
corr_polar_coords[:,1] = corr_polar_coords[:,1]-angle
corr_polar_vertices = polar_vertices.copy()
corr_polar_vertices[:,1] = polar_vertices[:,1]-angle

# Plot points, concave hull vertices, and rotated verticies
r = polar_coords[:, 0]
theta =  polar_coords[:, 1]
r_v = polar_vertices[:, 0]
theta_v = polar_vertices[:, 1]
corr_theta_v = corr_polar_vertices[:,1]
```

```
# Create polar plot
fig, ax = plt.subplots(subplot_kw={'projection': 'polar'})
ax.plot(theta, r, "bo")
#ax.plot(theta_v, r_v, "ro")
ax.plot(corr_theta_v, r_v, "go")
# Customize plot (optional)
ax.set_title("Concave hull vertices")
ax.grid(True)

plt.show()
```

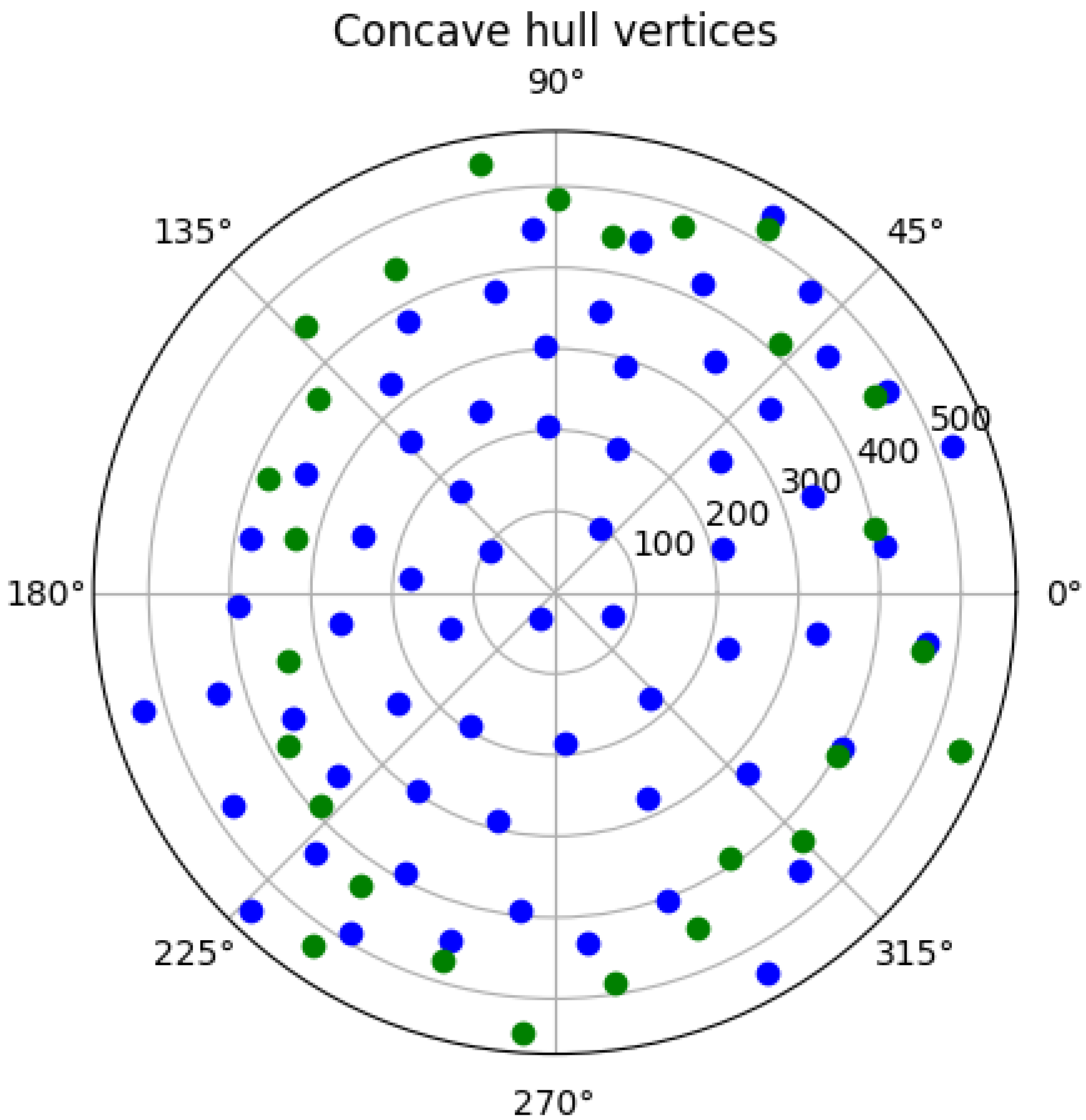

In [63]:
```
# Convert rotated polar points to cartesian
corr_coords = polar_to_cartesian(corr_polar_coords)

# Get convex hull
hull = c_hull(corr_coords)

# Get 360 boundary points and convert to polar
boundary = boundary_points(hull)
polar_boundary = cartesian_to_polar(boundary)

# Plot boundary and points on polar plot
r_b = polar_boundary[:, 0]
theta_b =  polar_boundary[:, 1]
r = corr_polar_coords[:, 0]
theta =  corr_polar_coords[:, 1]
```

Development • Supplementary information

```
# Create polar plot
fig, ax = plt.subplots(subplot_kw={'projection': 'polar'})
ax.plot(theta, r, "ro")
ax.plot(theta_b, r_b)
# Customize plot (optional)
ax.set_title("Convex Hull with Points")
ax.grid(True)

plt.show()
```

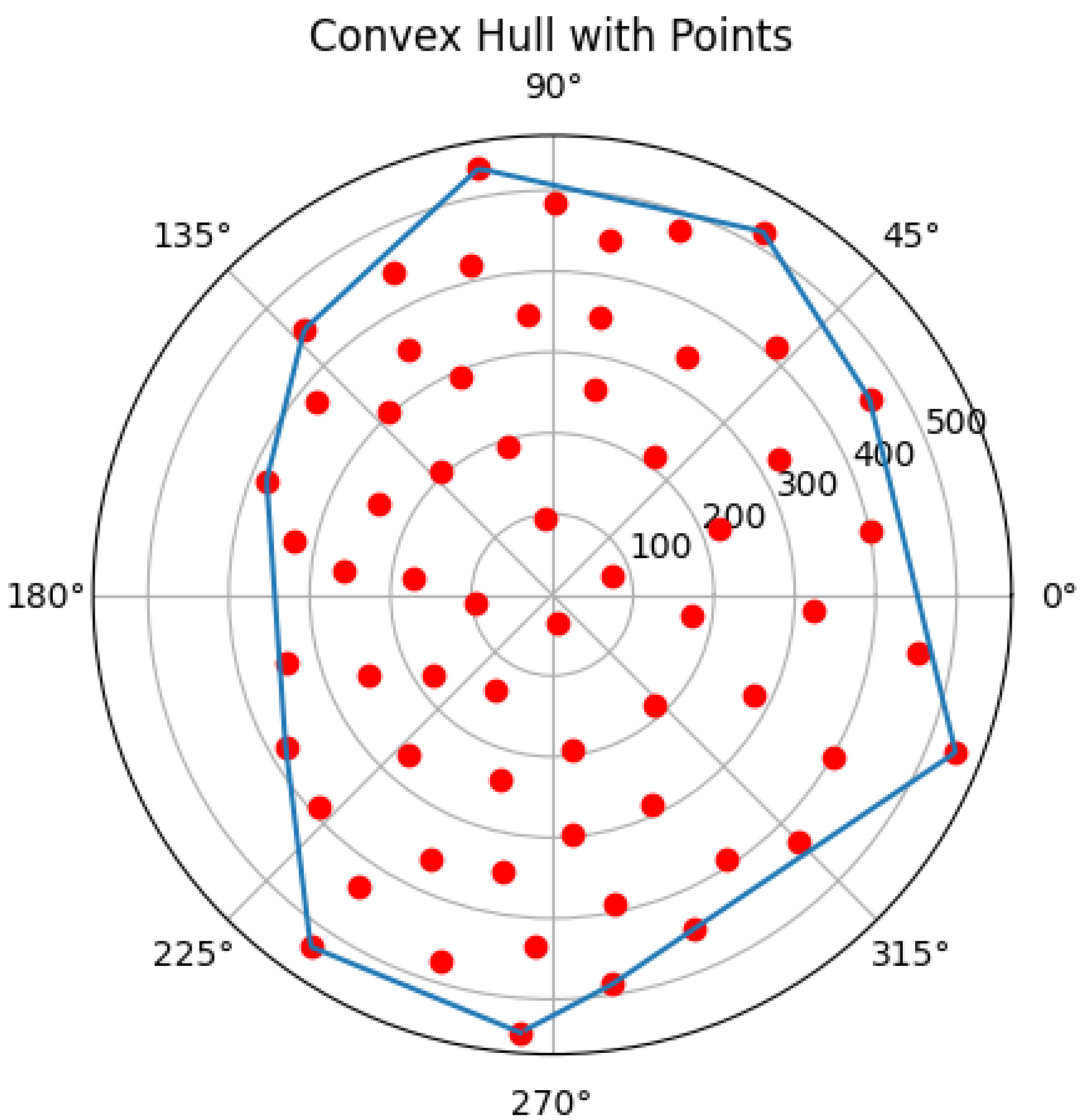

In [64]:
```
# Generate dict of scale factors for each degree using boundary points
factor_dict = make_factor_dict(polar_boundary)

# Get factors to test on convex hull
r_max = r_b.max()
r_norm = r_b/r_max
r_factor = r_max/r_b

# Check factors by creating polar plot of hull and hull expanded to circle
fig, ax = plt.subplots(subplot_kw={'projection': 'polar'})
ax.plot(theta_b, r_norm, 'b')
ax.plot(theta_b, r_factor*r_norm, 'r')
# Customize plot (optional)
ax.set_title("Hull Polar Plot")
ax.grid(True)

plt.show()
```

Development: doi:10.1242/dev.205363: Supplementary information

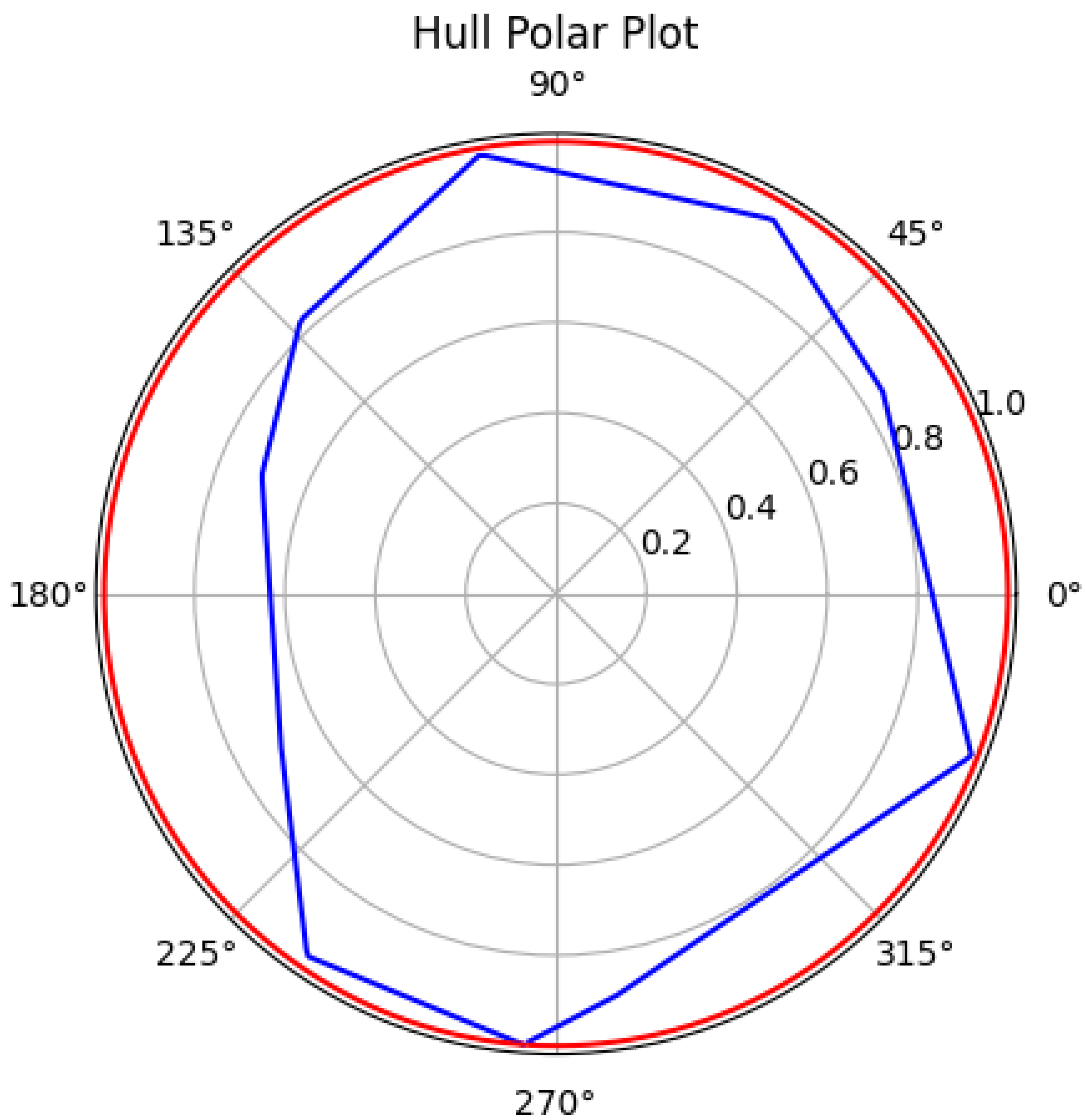

In [100…

```
# Alter r for each polar point using factor_dict and closest lookup
corr_polar_coords_n = corr_polar_coords.copy()
for i in range(len(corr_polar_coords[:,0])):
    corr_polar_coords_n[i,0] = closest_val(factor_dict, corr_polar_coords[i,

# Plot points and expanded points
r = corr_polar_coords[:, 0]
theta =  corr_polar_coords[:, 1]
r_n = corr_polar_coords_n[:, 0]
theta_n = corr_polar_coords_n[:, 1]

# Create polar plot
fig, ax = plt.subplots(subplot_kw={'projection': 'polar'})
#ax.plot(theta, r, "bo")
ax.plot(theta_n, r_n, "ro")
# Customize plot (optional)
ax.set_title("Point array (blue) normalized (red) to circle")
ax.grid(True)

plt.show()
```

Development • Supplementary information

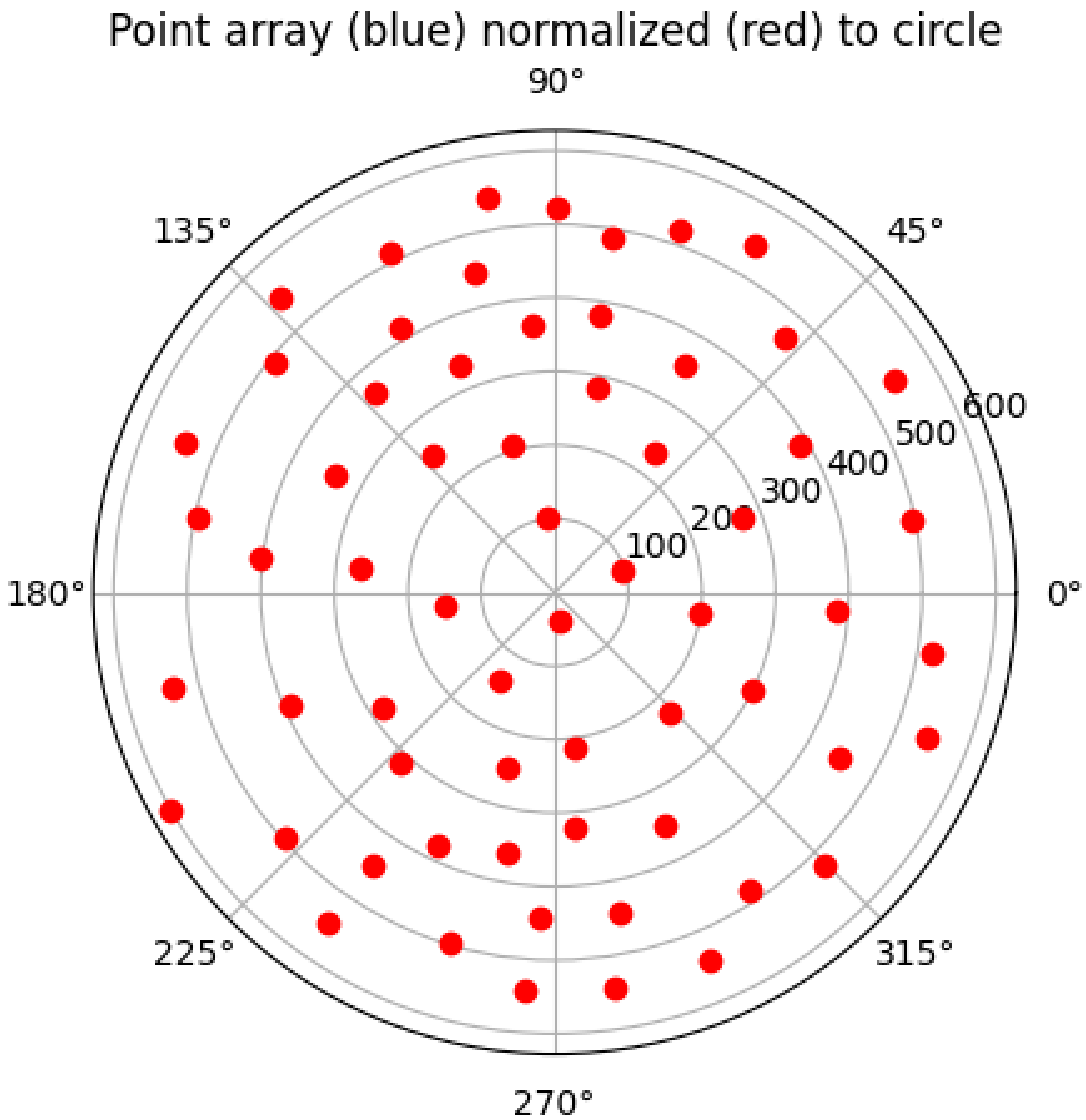

In [101…
```
# Update date table

merged_props['theta_n'] = theta_n
merged_props['r_n'] = r_n
```

Plot normalized points by expression

In [102…
```
# Classify cells
# change depending on experiment
def classify_cell(row):
    if row['red'] == 1 and row['far red'] == 1:
       return "Double+"
    elif row['red'] == 1:
        return "cabp1b+"
    elif row['far red'] == 1:
        return "cabp2b+"
    #else:
     #   return "Negative"

merged_props['marker_class'] = merged_props.apply(classify_cell, axis=1)

# Assign colors for each category
color_map = {
    "cabp1b+": "magenta",
    "cabp2b+": "gold",
    "Double+": "darkcyan",
```

```
    "Negative": "lime"
}
merged_props['color'] = merged_props['marker_class'].map(color_map)

# Plot normalized points in polar plot
fig, ax = plt.subplots(subplot_kw={'projection': 'polar'})
ax.scatter(theta_n, r_n, c=merged_props['color'])  # pass list of colors, no
ax.set_title("Polar plot by marker")
# Set 0 degrees to the top and clockwise direction
ax.set_theta_zero_location('N')  # 'N' puts 0° at the top
ax.set_theta_direction(1)       # counterclockwise
# label axes
angles = np.arange(0, 360, 45)
labels = ['Anterior', '45°', 'yellow', '135°', 'Posterior', '225°', 'pink',
ax.set_thetagrids(angles, labels=labels, fontsize = 14)

plt.show()
```

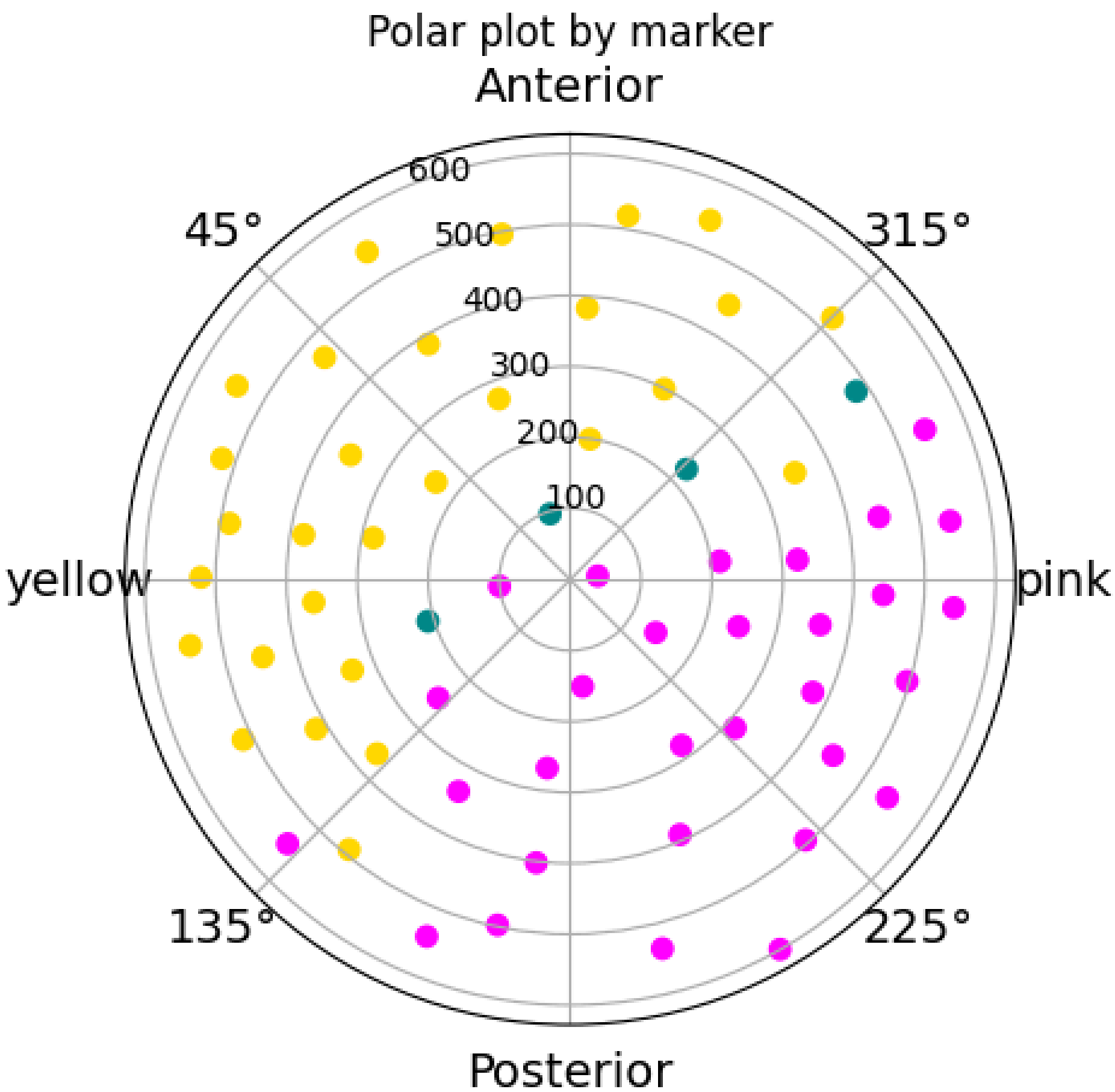

In [105…

```
## skip if 0-180 axis already delineates the striolar/extrastriolar boundary

## shift by x degrees
#shift_deg =  45
#shift_rad = np.radians(shift_deg)
#theta_n = (theta + shift_rad) % (2 * np.pi)

## or flip across the 90° line
axis_angle = np.pi/2 # mirror across 0 radians
theta_n = (2*axis_angle - theta_n) % (2*np.pi)
```

Development • Supplementary information

```
# To mirror across vertical axis, set axis_angle = 0

merged_props['theta_n'] = theta_n

# Plot normalized points in polar plot
fig, ax = plt.subplots(subplot_kw={'projection': 'polar'})
ax.scatter(theta_n, r_n, c=merged_props['color'])  # pass list of colors, no
ax.set_title("Polar plot by marker")

angles = np.arange(0, 360, 45)
labels = ['Anterior', '45°', 'yellow', '135°', 'Posterior', '225°', 'pink',

# Customize radial grid
ax.set_yticklabels([])

# Set 0 degrees to the top and clockwise direction
ax.set_theta_zero_location('N')  # 'N' puts 0° at the top
ax.set_theta_direction(1)       # counterclockwise
plt.show()
```

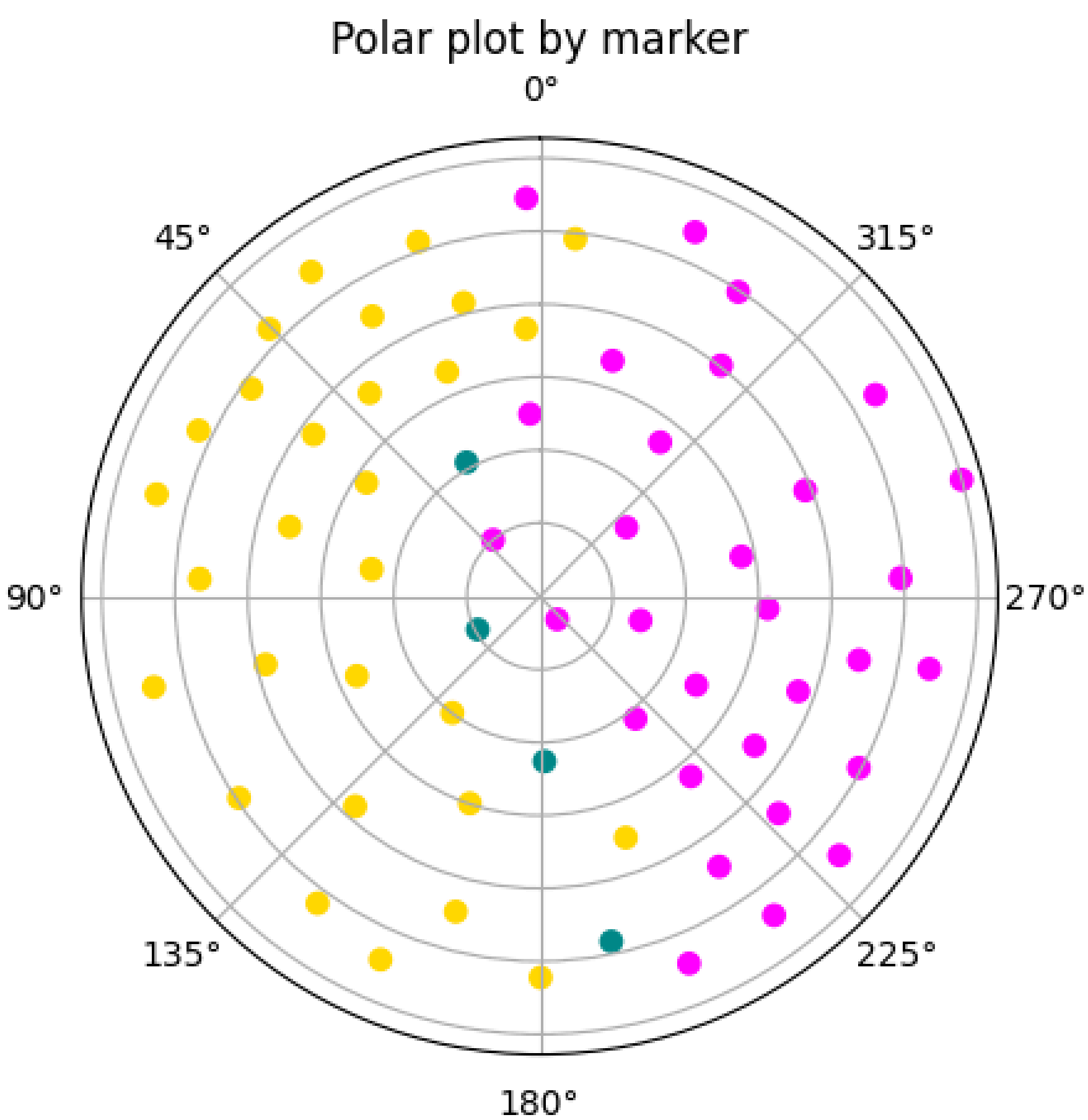

In [106…

```
estriola = merged_props[merged_props["marker_class"]== "cabp1b+"]

fig, ax = plt.subplots(subplot_kw={'projection': 'polar'})
ax.set_title("Extrastriolar HC", va='bottom', fontsize = 26, fontweight = 'b

# Set 0 degrees to the top and clockwise direction
ax.set_theta_zero_location('N')  # 'N' puts 0° at the top
```

```
ax.set_theta_direction(1)       # counterclockwise


# Plot data
ax.scatter(estriola['theta_n'], estriola['r_n'], c=estriola['color'], s=30)

# Customize radial grid
ax.set_yticklabels([])

# Define custom angular labels
angles = np.arange(0, 360, 45)
labels = ['A', ' ', 'L', ' ', 'P', ' ', 'M', ' ']
ax.set_thetagrids(angles, labels=labels, fontsize = 26)


plt.show()
```

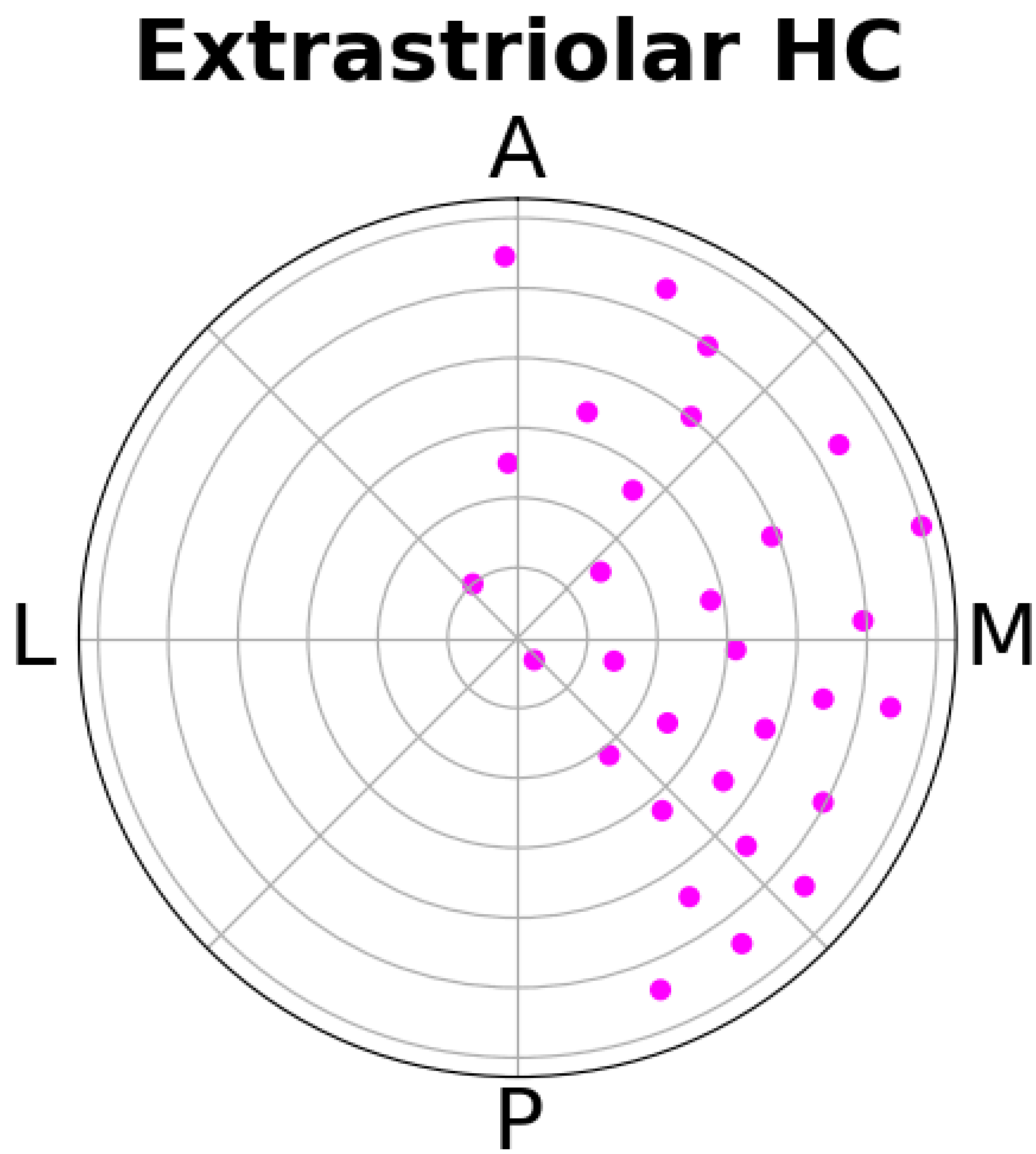

In [107…

```
striola = merged_props[merged_props["marker_class"]== "cabp2b+"]

fig, ax = plt.subplots(subplot_kw={'projection': 'polar'})
ax.set_title("Striolar HC", va='bottom', fontsize = 26, fontweight = 'bold')

# Set 0 degrees to the top and clockwise direction
ax.set_theta_zero_location('N')  # 'N' puts 0° at the top
ax.set_theta_direction(1)       # counterclockwise
```

Development • Supplementary information

```
# Plot data
ax.scatter(striola['theta_n'], striola['r_n'], c=striola['color'], s=30)  #

# Customize radial grid
ax.set_yticklabels([])

# Define custom angular labels
angles = np.arange(0, 360, 45)
labels = ['A', ' ', 'L', ' ', 'P', ' ', 'M', ' ']
ax.set_thetagrids(angles, labels=labels, fontsize = 26)


plt.show()
```

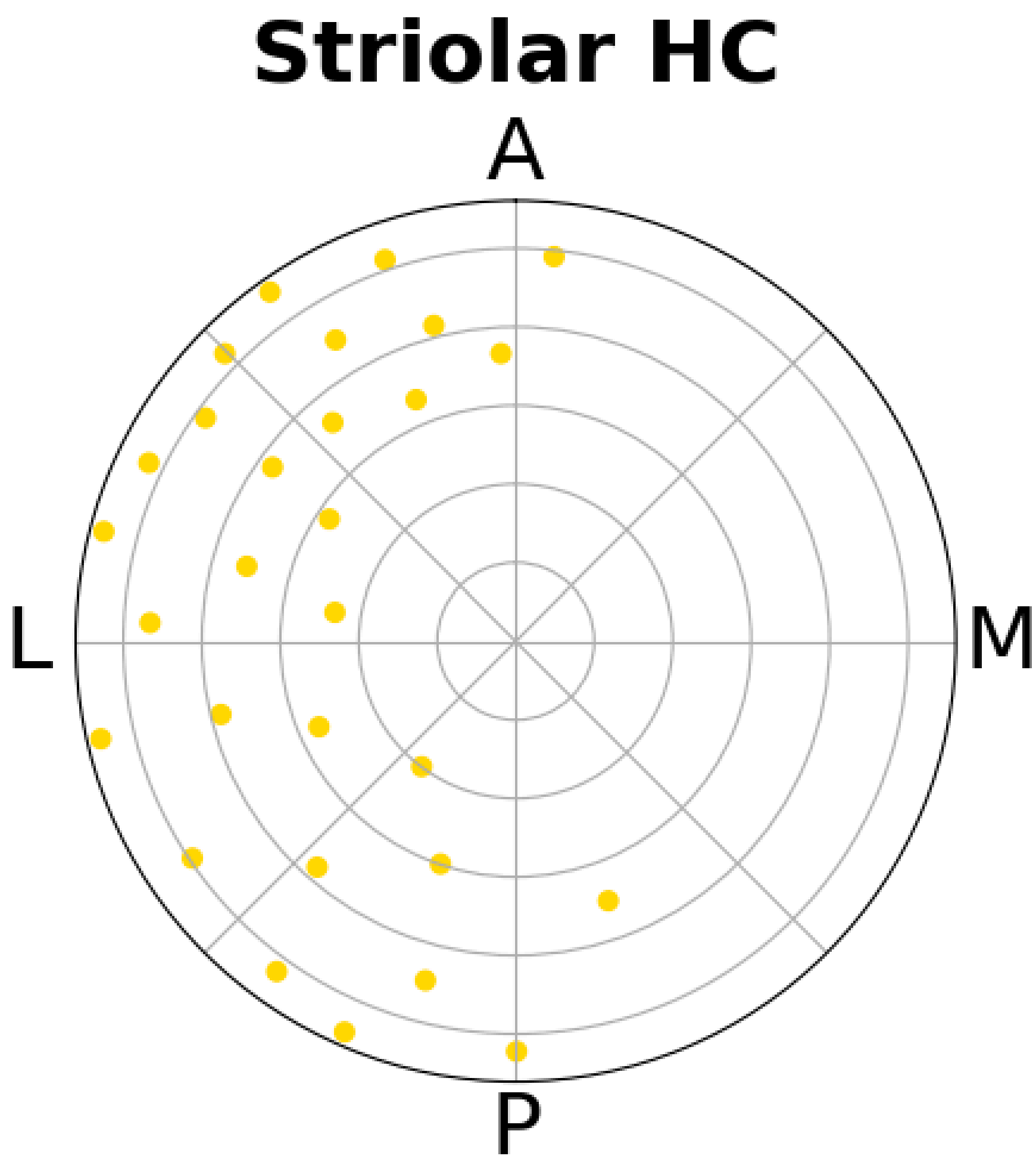

In [108…

```
double_labeled = merged_props[merged_props["marker_class"]== "Double+"]

fig, ax = plt.subplots(subplot_kw={'projection': 'polar'})
ax.set_title("Intermediate HC", va='bottom', fontsize = 26, fontweight = 'bo

# Set 0 degrees to the top and clockwise direction
ax.set_theta_zero_location('N')  # 'N' puts 0° at the top
ax.set_theta_direction(1)       # counterclockwise

# Plot data
ax.scatter(double_labeled['theta_n'], double_labeled['r_n'], c=double_labele
```

Development • Supplementary information

```
# Customize radial grid
ax.set_yticklabels([])

# Define custom angular labels
angles = np.arange(0, 360, 45)
labels = ['A', ' ', 'L', ' ', 'P', ' ', 'M', ' ']
ax.set_thetagrids(angles, labels=labels, fontsize = 26)


plt.show()
```

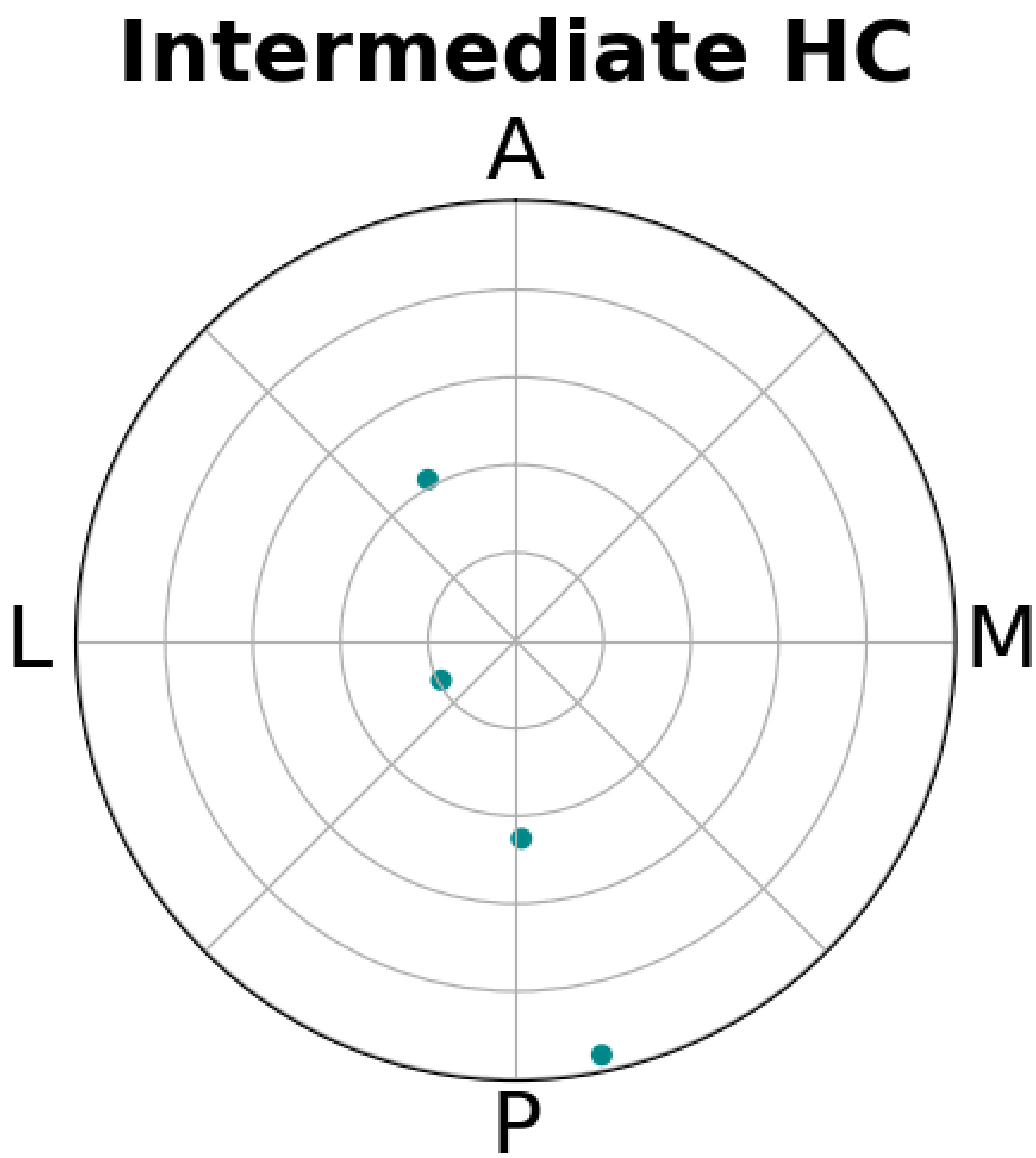

Save

```
merged_props.to_csv('merged_props.csv', index=False)
```
