# Supplementary material for: Activity and retinoic acid drive hair cell spatial patterning in the zebrafish utricle
Source: Development. 2026 Mar 23;153(6):dev205363. doi: 10.1242/dev.205363 (PMC13196668; doi:10.1242/dev.205363)
Supplement: Supplementary file 2 [file develop-153-205363_review_history.pdf]

Activity and retinoic acid drive hair cell spatial patterning in the zebrafish utricle

Selina Baeza-Loya, Jo Trang Bùi and David W. Raible
DOI: 10.1242/dev.205363

Editor: Ken Poss

Review timeline

Original submission:	11 November 2025
Editorial decision:	20 December 2025
First revision received:	9 January 2026
Accepted:	25 January 2026

Original submission

First decision letter

MS ID#: dev.205363

MS TITLE: Activity and retinoic acid drive hair cell spatial patterning in the zebrafish utricle

AUTHORS:Selina Baeza-Loya, Jo Trang Bùi and David W. Raible

Dear Dave,

I have now received all the referees reports on the above manuscript, and have reached a decision. The referees' comments are appended below.

The overall evaluation is positive and we would like to publish a revised manuscript in Development, provided that the referees' comments can be satisfactorily addressed. Please attend to all of the reviewers' comments in your revised manuscript and detail them in your point-by-point response. If you do not agree with any of their criticisms or suggestions explain clearly why this is so. If it would be helpful, you are welcome to contact us to discuss your revision in greater detail. Please send us a point-by-point response indicating your plans for addressing the referees' comments, and we will look over this and provide further guidance.

Reviewer 1

Advance summary and potential significance to field

Baeza-Loya and colleagues have submitted a manuscript characterizing the fate determination (striolar v. extrastriolar) of utricular hair cells in the early zebrafish larva. Using markers specific to the two types of hair cells, cabp1b for striolar cells and cabp2b for extrastriolar cells, they showed that the earliest emerging hair cells expressed both genes, but by 48h the utricle had primarily cabp1b expressing striolar cells. Over time the utricle then generated striolar and extrastriolar hair cells in roughly equal proportions with a zone in the middle with cells that expressed both markers. The enzymes that produce retinoic acid and degrade RA are asymmetrically expressed in the extrastriolar and striolar zones respectively. The authors show that treatment with retinoic acid or retinol alters the distribution of hair cell identities, essentially suppressing striolar fates while either promoting or stabilizing extrastriolar fates. They additionally showed that hair cell

© 2026. Published by The Company of Biologists under the terms of the Creative Commons Attribution License (https://creativecommons.org/licenses/by/4.0/).

mechanosensory transduction was also necessary for hair cells to commit to striolar or extrastriolar fates. This requirement appears to be independent of the RA gradient requirement.

Comments for the author

The experiments described in the manuscript are carefully executed and fairly clear to interpret. I do not have any objections to how the experiments were performed.
Minor comment:
I am wondering about the central zone where the hair cells possess an intermediate fate (express both makers). Is it known where new hair cells emerge when the sensory epithelium grows? In other words, are new cells added in the center of the tissue in an intermediate state and then the cells commit to either striolar or extrastriolar as the migrate into the appropriate field? This would mean that RA would be stabilizing the extrastriolar fate while the loss of RA would cause cells to take on the striolar fate.

<u>Reviewer 2</u>

Advance summary and potential significance to field

The manuscript from Baeza-Loya et al, titled, Activity and retinoic acid drive hair cell spatial patterning in the zebrafish utricle is interesting and timely. They used a wealth of data to show that zebrafish vestibular otolith organs develop distinct striolar and extrastriolar hair cell zones through a temporally regulated process. Early-born hair cells initially display mixed striolar and extrastriolar molecular features before resolving to an extrastriolar fate. Later-developing hair cells (>36 hours) mostly develop directly as striolar or extrastriolar. The authors identify spatially complementary retinoic acid (RA) signaling components associated with these zones and demonstrate that RA biases hair cell fate toward extrastriolar identity at the expense of striolar development. Importantly, in mechanotransduction-deficient mutants, normal RA patterning is insufficient to stabilize early hair cell fate, and RA treatment further disrupts zonal specification. Overall, the work supports a model in which both RA signaling and hair cell activity are required for proper vestibular hair cell zonal patterning.

Strengths
* The figures are clear and effective.
* The data are multimodal, integrating RNA-seq, HCR, retinoic acid (RA) manipulations, and other approaches.
* The manuscript is well written.
* The findings are exciting and of broad interest.

Clarifications
* It is unclear why the authors refer to the anterior macula and posterior macula in Figure 3A, rather than the utricle and saccule. Is this difference in terminology due to developmental stage or age differences between the samples shown in Figures 3A and 3B?

Sincerely,
Brian M. McDermott, Jr.

Comments for the author

None

<u>Reviewer 3</u>

Advance summary and potential significance to field

In the manuscript "Activity and retinoic acid drive hair cell spatial patterning in the zebrafish utricle" the authors build upon previously known insights on how zonal identity is determined in the utricle. While the existence of molecular differences between hair cells in striolar and extrastriolar

© 2026. Published by The Company of Biologists under the terms of the Creative Commons Attribution License (https://creativecommons.org/licenses/by/4.0/).

zones and the role of retinoic acid signaling in determining these zones was previously known in mammals, this paper presents new findings in 1) how zonal fate is established in early hair cell development 2) how RA gradients are established and how RA signaling affects hair cell fate and 3) how mechanotransduction influences maturation of spatial patterning. These results are of broad interest to the readers of Development and are broadly important to the field of hair cell biology.

Comments for the author

I found the results in the paper to be generally clear and supportive of the conclusions presented in the paper, however I believe the paper could be improved by the following changes.
1. A broad criticism of this paper is the lack of detail in the figure legends, which at times complicated interpretation of the data. While some of the details were included in the methods, it is easier for readers to follow when the information is presented directly alongside the data. Please include the following in figure legends: what error bars represent in all graphs, what the N represents in all graphs (number of fish, number of utricles, number of hair cells), p values where significance is represented.
2. Figure 2: from the presented image, there are segmented hair cells that are in areas without GFP signal that is easily visible. Were any pre-processing steps done to enable segmentation in these cases? I am assuming that the myo6b-GFP signal was used for segmentation though it was not explicitly stated in the methods.
3. It would also be helpful to describe more in depth what cutoffs were used for categorizing cells as positive or negative for FISH markers. The authors state that mean or sum across pixels was used but provide no further information on how cut offs were determined, and whether the same cut off was used across all images or if different values were used for individual images. These should be explicitly stated for the sake of reproducibility and understanding the reliability of the analysis metric. Since many conclusions of the paper rely on determining the proportion of cells falling into categories based on this thresholding of FISH signal, this is an important point. Unfortunately at the time of this review the link to the analysis code deposited on GitHub did not work, and thus I am unable to review the provided code to determine if it helps answer these questions.
4. I found the visual presentation of the photoconverted data to be confusing. I am assuming that 4 color imaging was not technically possible for the authors, so the photoconverted cells are also in the same channel as one of the HCR labels. I think this was also the motivation for the switch in labeling colors in Figure 4. I think it might be beneficial to readers for some of this to be explicitly stated to aid in interpretation of the images. This is also a case where explicit description of the methods used to discriminate between photoconverted cells and cells with strong HCR signal may be useful. The methods suggests that these cells were manually segmented, however, authors could still include criteria used to make a segmentation call.
5. The authors acknowledge that use of a loss of function approach would strengthen the conclusions in the paper, as much of the conclusions rely on visualization of transcript expression. Another suggestion might be overexpression of aldh1a3 or cyp26b1 in support cells to test whether you can create intermediate type hair cells outside of the medial region of the utricle by creating new zones of intermediate RA signaling. While possibly too technically challenging, direct visualization of RA gradients is also possible with RA biosensors such as GEPRA. Use of an additional approach may strengthen the conclusions of this paper.

First revision

<u>Author response to reviewers' comments</u>

We are grateful for the Reviewers' time, effort, and support. We've carefully considered all the Reviewers' comments, suggestions, and questions. We have made improvements to the Methods section detailing the computational pipeline, particularly regarding the thresholding parameters, and to the figure legends, which improves comprehension of the data. We believe this improves the manuscript.

© 2026. Published by The Company of Biologists under the terms of the Creative Commons Attribution License (https://creativecommons.org/licenses/by/4.0/).

Reviewer 1:

Minor comment:

I am wondering about the central zone where the hair cells possess an intermediate fate (express both makers). Is it known where new hair cells emerge when the sensory epithelium grows? In other words, are new cells added in the center of the tissue in an intermediate state and then the cells commit to either striolar or extrastriolar as the migrate into the appropriate field? This would mean that RA would be stabilizing the extrastriolar fate while the loss of RA would cause cells to take on the striolar fate.

Thanks for the question. The few experiments we did on tracking hair cell addition (not shown) in the utricle indicated that, during larval stages, hair cells are only added at the periphery in a ring-like fashion. While we did not exhaustively investigate this, newly added hair cells on the periphery do not seem to transition from intermediate to striolar/extrastriolar, as they are in RA rich or deficient environment.

Reviewer 2:

Clarifications

* It is unclear why the authors refer to the anterior macula and posterior macula in Figure 3A, rather than the utricle and saccule. Is this difference in terminology due to developmental stage or age differences between the samples shown in Figures 3A and 3B?

We used the anterior and posterior macula here to conform with the previous literature. We clarify this point (lines 198-199) but have adjusted the figure to keep the nomenclature consistent.

Reviewer 3

1. A broad criticism of this paper is the lack of detail in the figure legends, which at times complicated interpretation of the data. While some of the details were included in the methods, it is easier for readers to follow when the information is presented directly alongside the data. Please include the following in figure legends: what error bars represent in all graphs, what the N represents in all graphs (number of fish, number of utricles, number of hair cells), p values where significance is represented.

Thank you for the feedback. More information regarding graphs and p-values has been added to the figure legends.

2. Figure 2: from the presented image, there are segmented hair cells that are in areas without GFP signal that is easily visible. Were any pre-processing steps done to enable segmentation in these cases? I am assuming that the myo6b-GFP signal was used for segmentation though it was not explicitly stated in the methods.

We now include separate channels so that it is more apparent that instances segmented by the Cellpose-SAM algorithm correspond to *myo6b:GFP*. In addition, we clarify that this signal was used for segmentation in the figure legend (line 826).

3. It would also be helpful to describe more in depth what cutoffs were used for categorizing cells as positive or negative for FISH markers. The authors state that mean or sum across pixels was used but provide no further information on how cut offs were determined, and whether the same cut off was used across all images or if different values were used for individual images. These should be explicitly stated for the sake of reproducibility and understanding the reliability of the analysis metric. Since many conclusions of the paper rely on determining the proportion of cells falling into categories based on this thresholding of FISH signal, this is an important point.

We provide additional detail for our methods determining threshold using the Fisher-Jenks natural breaks algorithm (lines 148-149).

© 2026. Published by The Company of Biologists under the terms of the Creative Commons Attribution License (https://creativecommons.org/licenses/by/4.0/).

Unfortunately at the time of this review the link to the analysis code deposited on GitHub did not work, and thus I am unable to review the provided code to determine if it helps answer these questions.

We apologize for the problem with access to the GitHub site. We now include code as a supplemental file in addition to the GitHub link.

4. I found the visual presentation of the photoconverted data to be confusing. I am assuming that 4 color imaging was not technically possible for the authors, so the photoconverted cells are also in the same channel as one of the HCR labels. I think this was also the motivation for the switch in labeling colors in Figure 4. I think it might be beneficial to readers for some of this to be explicitly stated to aid in interpretation of the images. This is also a case where explicit description of the methods used to discriminate between photoconverted cells and cells with strong HCR signal may be useful. The methods suggests that these cells were manually segmented, however, authors could still include criteria used to make a segmentation call.

It is correct that we could only work in three color channels. Thus, the photoconversion overlapped with one HCR signal. However, the characteristic of the signal (smooth, uniform nuclear color vs cytoplasmic puncta) is sufficiently different that we were able to distinguish between the two. We have clarified the color switch in the figure legend (lines 852-854) and also provided additional detail for our methods of distinguishing photoconverted signal from HCR puncta (lines 149-151).

5. The authors acknowledge that use of a loss of function approach would strengthen the conclusions in the paper, as much of the conclusions rely on visualization of transcript expression. Another suggestion might be overexpression of aldh1a3 or cyp26b1 in support cells to test whether you can create intermediate type hair cells outside of the medial region of the utricle by creating new zones of intermediate RA signaling. While possibly too technically challenging, direct visualization of RA gradients is also possible with RA biosensors such as GEPRA. Use of an additional approach may strengthen the conclusions of this paper.

We agree with the reviewer that additional studies will be needed to clarify the roles of RA in patterning. We have included additional discussion of this point in the Study Limitations section of the Discussion (lines 537-540).

<u>Second decision letter</u>

MS ID#: dev.205363R1

MS TITLE: Activity and retinoic acid drive hair cell spatial patterning in the zebrafish utricle

AUTHORS:Selina Baeza-Loya, Jo Trang Bùi and David W. Raible

Dear Dave,

I am happy to tell you that your manuscript has been accepted for publication in Development, pending our standard publication integrity checks.

<u>Reviewer 3</u>

Advance summary and potential significance to field

The revisions to the manuscript have improved readability and clarified experimental methods. Thank you for your efforts, and well done on an interesting story!

© 2026. Published by The Company of Biologists under the terms of the Creative Commons Attribution License (https://creativecommons.org/licenses/by/4.0/).
